# A solvent-free solid catalyst for the selective and color-indicating ambient-air removal of sulfur mustard

**DOI:** 10.1038/s42004-021-00465-7

**Published:** 2021-03-08

**Authors:** Daniel L. Collins-Wildman, Kevin P. Sullivan, Yurii V. Geletii, Victoria G. Snider, Wesley O. Gordon, Alex Balboa, Yiyao Tian, Rachel M. Slaugenhaupt, Alexey L. Kaledin, Christopher J. Karwacki, Anatoly I. Frenkel, Djamaladdin G. Musaev, Craig L. Hill

**Affiliations:** 1 Department of Chemistry, Emory University, Atlanta, GA 30322 USA; 2 U.S. Army Combat Capabilities Development Command Chemical Biological Center, Aberdeen, MD 21010 USA; 3 Department of Materials Science and Chemical Engineering, Stony Brook University, Stony Brook, NY 11794 USA; 4 Cherry L. Emerson Center for Scientific Computation, Emory University, Atlanta, GA 30322 USA; 5 Chemistry Division, Brookhaven National Laboratory, Upton, NY 11973 USA

**Keywords:** Catalyst synthesis, Solid-state chemistry, Pollution remediation, Sensors and biosensors

## Abstract

Bis(2-chloroethyl) sulfide or sulfur mustard (HD) is one of the highest-tonnage chemical warfare agents and one that is highly persistent in the environment. For decontamination, selective oxidation of HD to the substantially less toxic sulfoxide is crucial. We report here a solvent-free, solid, robust catalyst comprising hydrophobic salts of tribromide and nitrate, copper(II) nitrate hydrate, and a solid acid (Nafion^TM^) for selective sulfoxidation using only ambient air at room temperature. This system rapidly removes HD as a neat liquid or a vapor. The mechanisms of these aerobic decontamination reactions are complex, and studies confirm reversible formation of a key intermediate, the bromosulfonium ion, and the role of Cu(II). The latter increases the rate four-fold by increasing the equilibrium concentration of bromosulfonium during turnover. Cu(II) also provides a colorimetric detection capability. Without HD, the solid is green, and with HD, it is brown. Bromine K-edge XANES and EXAFS studies confirm regeneration of tribromide under catalytic conditions. Diffuse reflectance infrared Fourier transform spectroscopy shows absorption of HD vapor and selective conversion to the desired sulfoxide, HDO, at the gas–solid interface.

## Abstract (web summary)

Decontamination of sulfur-containing chemical warfare agents can be achieved through selective, air-based oxidation. Here a solid, solvent-free catalyst for aerobic oxidative decontamination of sulfur mustard is reported.

## Introduction

The selective conversion of sulfides to sulfoxides without overoxidation to the sulfone is a key synthetic reaction for multiple applications.^1–6^ One particularly pressing application is the decontamination of bis(2-chloroethyl) sulfide (sulfur mustard or HD), one of the highest tonnage chemical warfare agents (CWA) and one that is highly persistent in the environment.^7–12^ While HD can be removed, in principle, by either oxidation or hydrolysis, oxidation is generally targeted as the hydrolysis reaction with HD is quite slow.^13^ Dioxygen represents an ideal oxidant as it is abundant, inexpensive, and atom economical.^14,15^ For oxidative decontamination, selective oxidation of HD to sulfoxide (Eq. 1) is crucial as the sulfoxide is substantially less toxic than the more oxidized sulfone.^9,10,13,16–18^ Materials capable of selective O_2_-based sulfoxidation are therefore highly desirable.^1,2,19,20^ Specifically, there is a need to develop catalytic systems capable of rapid and selective decontamination of HD under ambient conditions.

1 $$\left( {{\mathrm{ClCH}}_2{\mathrm{CH}}_2} \right)_2{\mathrm{S}} + \raise.5ex\hbox{$\scriptstyle 1$}\kern-.1em/ \kern-.15em\lower.25ex\hbox{$\scriptstyle 2$} {\mathrm{O}}_2 \to \left( {{\mathrm{ClCH}}_2{\mathrm{CH}}_2} \right)_2{\mathrm{SO}}$$

Given their selective sulfoxidation activity in solution, many studies have explored systems containing bromine (Br_*y*_) and nitrogen oxide (NO_*x*_) species.^21–24^ More recently, this type of system was proven effective for sulfoxidation of the HD simulant, 2-chloroethyl ethyl sulfide (CEES), in acetonitrile.^25–27^ However, despite promising results, (a) solid formulations of these oxidation catalysts to enable practical applications are unknown, (b) fundamental aspects of the complicated mechanism have eluded researchers, and (c) live agent (HD) studies are lacking. Understanding how this promising system behaves with live agent and in the absence of additional solvent, is crucial to the development of a truly effective material for catalytic aerobic HD removal.

Here, we report the development of such a material working as a solvent-free, solid-formulation catalyst (henceforth SFC) for selective air (O_2_)-based sulfoxidation of both live agent HD and its simulant, CEES. SFC comprises tetrabutylammonium tribromide (TBABr_3_), tetrabutylammonium nitrate (TBANO_3_), cupric nitrate trihydrate (Cu(II)), and Nafion^TM^. We were able to formulate this effective solid material for HD decontamination by first addressing key aspects of the proposed complex reaction mechanism outlined in Supplementary Fig. 1. These insights included demonstration of an equilibrium associated with the formation of a key reactive intermediate and the effects of Cu(II) on the catalytic system. In addition, through the use of X-ray absorption spectroscopy, we were able to follow the regeneration of the catalytic component, tribromide, during a solvent-free reaction with CEES.

## Results

### Mechanistic studies in acetonitrile

**Fig. 1:**
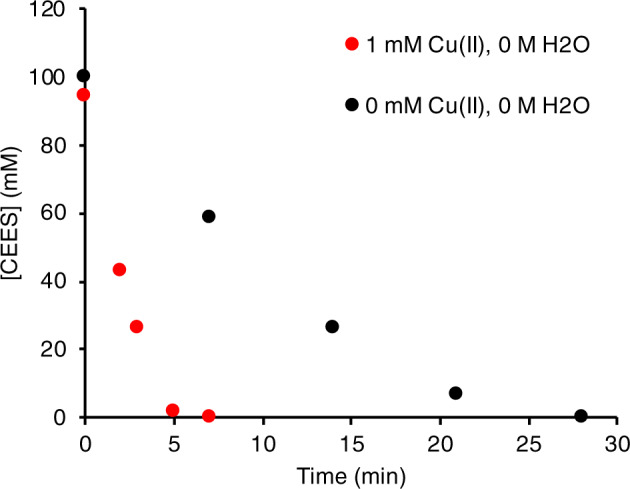
Kinetics of CEES oxidation by O_2_ catalyzed by Br_*y*_/NO_*x*_ with and without Cu(II). Conditions: 5.0 mM TBABr_3_, 10 mM *p*-TsOH, 10 mM TBANO_3_, 103 mM CEES, 70 mM 1,3-dichlorobenzene (1,3-DCB) internal standard, in MeCN under 1 atm. of air at ambient temperature (~22 °C). Red curve = 1.0 mM Cu(ClO_4_)_2_•6H_2_O, black curve = 0 mM Cu(ClO_4_)_2_•6H_2_O. These curves represent 10 turnovers based on NO_3_^−^.

The combination of tribromide and nitrate effectively catalyzes sulfoxidation reactions including that of the mustard simulant CEES.^25,27^ Studies have shown that transition metals can have significant effects in catalyzing the oxidation of sulfides.^28–32^ We demonstrate here a significant acceleration in the rate of sulfoxidation in the presence of Cu(II). The addition of 1.0 mM Cu(II) to the reaction solution of 5.0 mM Br_3_^−^ and 10 mM NO_3_^−^ results in an initial rate for aerobic sulfoxidation roughly 4 times faster than that without copper (Fig. 1). This corresponds to the decontamination of 10 equivalents of simulant in under 7 min, which represents, to our knowledge, the fastest catalytic system for selective aerobic sulfoxidation. Importantly, the reaction remains selective in the presence of Cu(II) and quantitatively produces the desired sulfoxide product as confirmed by ^13^C NMR (Supplementary Fig. 2).

To elucidate the role of copper in enhancing the rate of sulfide oxidation, we must first examine certain aspects of the proposed complex catalytic cycle outlined in Supplementary Fig. 1. The first step involves the reaction between sulfide, CEES, and bromine to form a bromosulfonium complex that is likely a reaction intermediate.^6,26,33,34^

2 $${\mathrm{R}}_2{\mathrm{S}} + {\mathrm{Br}}_2 \rightleftarrows {\mathrm{R}}_2{\mathrm{S}}^ + {\mathrm{Br}} + {\mathrm{Br}}^ -$$

Due to the equilibrium shown in Eq. 3, tribromide solutions will contain both Br_3_^−^ and some Br_2_ and Br^−^ with reported equilibrium constants of *K* = 17 and 9 × 10^6^ M^−1^ in water and in acetonitrile, respectively.^35–37^ Solutions of Br_3_^−^ and Br_2_ both react with sulfides to form the bromosulfonium intermediate (Supplementary Fig. 3). In the case of tribromide, it is generally predicted that the Br_3_^−^ ion acts as a reservoir for Br_2_ (via the equilibrium in Eq. 3), which is ultimately the reactive species toward to the sulfide.^25–27^

3 $${\mathrm{Br}}^ - + {\mathrm{Br}}_2 \rightleftarrows {\mathrm{Br}}_3^ -$$

The bromosulfonium intermediate (Eq. 2) is proposed to form the sulfoxide via oxidation by NO_3_^−^.^25,27^ Therefore, shifts in the equilibrium associated with the formation of this bromosulfonium complex will have a significant impact on the overall reaction rate if the bromosulfonium species is prior to or in the rate limiting step. The selectivity for the sulfoxide product (2-chloroethyl ethyl sulfoxide, CEESO) over the sulfone (CEESO_2_) product arises because the reactive bromosulfonium intermediate cannot be formed from the sulfoxide and therefore further oxidation to CEESO_2_ cannot occur via this pathway. Stopped-flow UV-Vis measurements at 446 nm (the isosbestic point for Br_2_/Br_3_^−^)^38^ allow quantification of the total Br_2_/Br_3_^−^ over time. For a solution of 5.0 mM tribromide and 50 mM CEES, this equilibrium is shifted in favor of the reactants (Br_3_^−^ and sulfide), however, the addition of 1–4% H_2_O (v/v) shifts the equilibrium in favor of the bromosulfonium complex (Supplementary Fig. 4). After the loss of Br_2_/Br_3_^−^ upon reaction with CEES in the presence of H_2_O, the desiccant MgSO_4_ can be added to remove the water in the system. As the water is absorbed by MgSO_4_, Br_2_/Br_3_^−^ are gradually regenerated demonstrating the truly reversible nature of Eq. 2 (Supplementary Fig. 5). Water is likely shifting this equilibrium by slowing the reverse reaction between the bromosulfonium cation and bromide. This leads to higher concentrations of products in the presence of water compared with higher concentrations of reactants in the absence of water.

Similar to water, addition of Cu(II) causes an equilibrium shift in favor of the bromosulfonium complex (Supplementary Fig. 6). In this case, the reaction is followed by the growth of CuBr_3_^−^, which has a ligand-to-metal charge transfer (LMCT) band at 635 nm.^39,40^ This can be used as an indirect means to monitor the concentration of Br_2_/Br_3_^−^ as the Br^−^ liberated upon the forward reaction between sulfide and Br_2_/Br_3_^−^ in Eq. 2 quickly forms complexes with Cu (Eq. 4).

4 $${\mathrm{Cu}}^{2 + } + x{\mathrm{Br}}^ - \rightleftarrows \left( {{\mathrm{CuBr}}_x} \right)^{(2 - x)}$$

Given the lack of absorption at 635 nm in the absence of sulfide (Supplementary Fig. 6), the rapid formation of CuBr_3_^−^ directly correlates with the formation of the bromosulfonium intermediate. For copper, the equilibrium in Eq. 2 again shifts in favor of the bromosulfonium intermediate as the reverse reaction with Br^−^ is inhibited by the complexation of free Br^−^ ions by copper. As shown in Fig. 1 the presence of Cu(II) clearly enhances the overall rate, which is consistent with an increase in the concentration of the reactive bromosulfonium intermediate.

Given the strong effect of copper, we also examined the effect of Zn(BF_4_)_2_ (Zn(II)) to see if a similar equilibrium shift would occur. As with copper, zinc readily forms complexes with Br^−^ ions (Eq. 5).^41^

5 $$Zn^{2 + } + x{\mathrm{Br}}^ - \rightleftarrows \left( {{\mathrm{ZnBr}}_x} \right)^{(2 - x)}$$

In this case, however, the complexes are colorless allowing the reaction to be followed by the loss of Br_2_/Br_3_^−^ at 446 nm. In the presence of 5.0 mM of Zn(II), the majority of Br_2_/Br_3_^−^ is consumed in under 10 s indicating a rapid equilibrium shift of Eq. 2 in favor of the products (Supplementary Fig. 7). As with copper this is likely caused by decreasing the rate of the reverse reaction due to the lower concentration of free Br^−^. To confirm this, we added 10 mM Br^−^ to the initial Zn solution mixed with CEES and observed that these additional Br^−^ ions did in fact lessen the effect of Zn (Supplementary Fig. 8).

Interestingly, while Cu(II), Zn(II), and H_2_O all shift the initial reaction between Br_2_ and sulfide in favor of the bromosulfonium intermediate, only Cu shows an enhancement in the overall rate of catalysis. For reactions containing 1.0 M water, the overall sulfoxidation is similar to the rate in the absence of water. For reactions containing both copper and water, the presence of 1.0 M water slows down the reaction (Supplementary Fig. 9). In the presence of 5.0 mM Zn(II), the reaction is also slowed. This likely occurs as the presence of water and zinc slow the oxidation of Br^−^ ions back to Br_2_, which completes the catalytic cycle for Br−containing species. One of the anticipated reduced NO_*x*_ species formed during the catalytic cycle (Supplementary Fig. 1) is nitrous acid, which is known to oxidize Br^−^ to Br_2_ via the following equilibria (Eqs. 6 and 7).^42,43^

6 $${\mathrm{HNO}}_2 + {\mathrm{Br}}^ - + {\mathrm{H}}^ + \rightleftarrows {\mathrm{NOBr}} + {\mathrm{H}}_2{\mathrm{O}}$$

7 $$2{\mathrm{NOBr}} \rightleftarrows 2{\mathrm{NO}} + {\mathrm{Br}}_2$$

Stopped-flow UV-Vis measurements show that increasing concentrations of water or zinc greatly inhibit the oxidation of Br^−^ by nitrous acid (Supplementary Figs. 10 and 11). In contrast, the oxidation of Br^−^ to Br_2_ proceeds similarly in the presence of Cu(II) ions compared to the control (Supplementary Fig. 12). This suggests that copper, unlike water or zinc, is able to shift the equilibrium of the initial reaction between sulfide and Br_2_/Br_3_^−^ without disrupting other components of the catalytic system thereby affording a net increase in the overall rate.

In addition to markedly improving the rate of catalysis, the use of Cu(II) also enables the colorimetric detection of sulfides in the system. Immediately upon exposure to CEES, the catalytic solution undergoes a dramatic color change from pale yellow to dark green, attributable to the formation of CuBr_3_ (Supplementary Fig. 13).^39,40^ Upon reaction completion, Br^−^ ions are no longer produced from the reaction between Br_2_ and CEES allowing all of the Br^−^ ions to be oxidized re-establishing the original Br_3_^−^ concentration (via Eqs. 6, 7, and 2). This returns the solution to its original yellow color and thereby indicates when the sulfide (HD or simulant CEES) has been fully decontaminated.

### The solid, color-indicating, aerobic mustard (HD) oxidation catalyst

With the above solution catalytic studies in hand, we turned to formulating a solid version of this catalyst to enable the development of protective materials (garments, masks, coatings, etc.) for removal/colorimetric detection of HD. Informed by previous studies,^26,27^ and our own work with solution-phase reactions, we incorporated sources of acid and copper along with tribromide and nitrate. Nafion^TM^ was chosen for the acid as it is a chemically robust perfluorinated polymer, and it is well tolerated by human skin. By utilizing Cu(NO_3_)_2_•3H_2_O, we were able to incorporate both a source of copper and nitrate. Through the use of quaternary ammonium salts of nitrate and tribromide, the active catalytic components are stable under ambient conditions and readily dissolve in the sulfide, providing a solid formulation that reacts directly with live agent in the absence of solvent. The end result is a fully selective, color-indicating solvent-free, solid catalyst (SFC) comprising a 5.0:3.3:1.7:2.3 mole ratio of TBABr_3_, TBANO_3_, Cu(NO_3_)_2_•3H_2_O, and Nafion^TM^ polymer, respectively (moles of Nafion^TM^ reported as equivalents of H^+^).

**Fig. 2:**
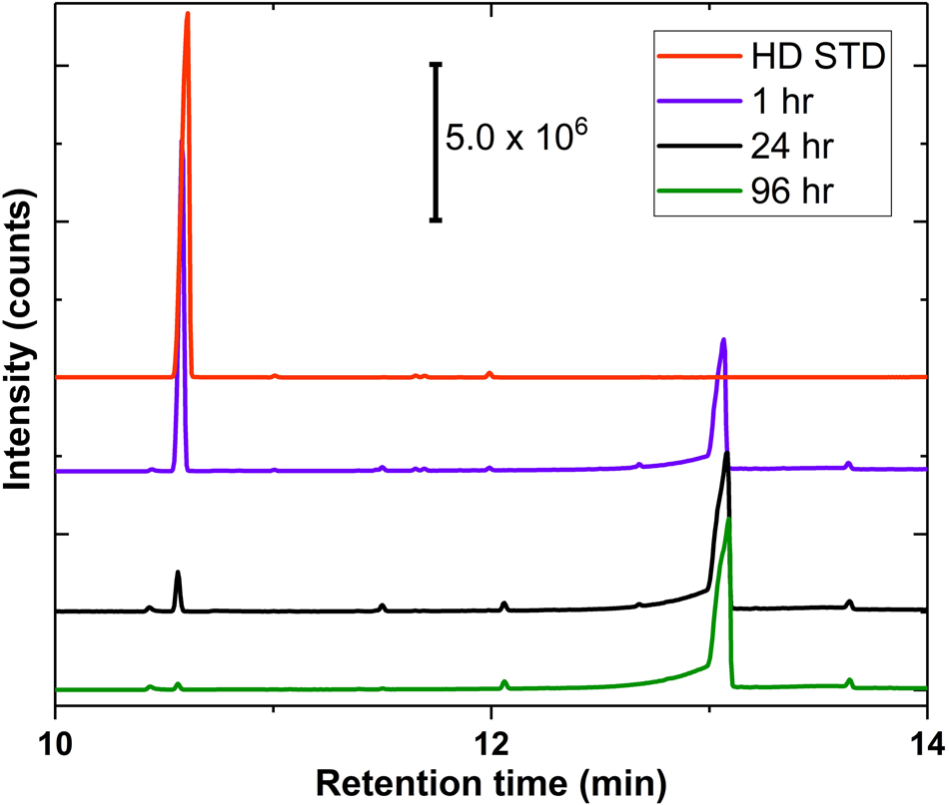
GC-MS spectra of live agent HD oxidation kinetics catalyzed by **SFC**. Conditions: 5 μL HD added directly to 5 mg SFC (10 turnovers based on Br_3_^−^). Reaction conducted in sealed vial with 20 mL syringe filled with O_2_ as gas headspace at ambient temperature (~22 °C). GC-MS measurements taken via 1.5 mL CDCl_3_ extraction. The peak intensities, which are the counts on the *y* axis, are normalized for each spectrum, i.e., the baselines are adjusted and different for each spectrum.

To assess the effectiveness of SFC in the absence of additional solvent, liquid aliquots of sulfide were placed directly on this solid catalyst. Upon exposure to 22 equivalents of neat CEES (relative to the Br_3_^−^ in SFC), SFC completely and selectively catalyzes production of the sulfoxide using only oxygen in ambient air as the terminal oxidant at ambient temperature (Supplementary Figs. 14 and 15). Exposure of SFC to two common battlefield contaminants, octane (as a surrogate for gasoline) and CO_2_, did not show any measurable inhibition of the reaction rate (Supplementary Fig. 16). Significantly, selective aerobic sulfoxidation was also observed with 10 equivalents of live agent HD (Fig. 2 and Supplementary Fig. 17). This catalytic system thus represents a significant advance in the development of protective materials against HD, as it is capable of decontaminating HD selectively and catalytically upon contact. Finally, this system is also colorimetric, immediately revealing distinct color changes from light green to dark brown in the presence of the harmful agent/simulant (Supplementary Fig. 18).

**Fig. 3:**
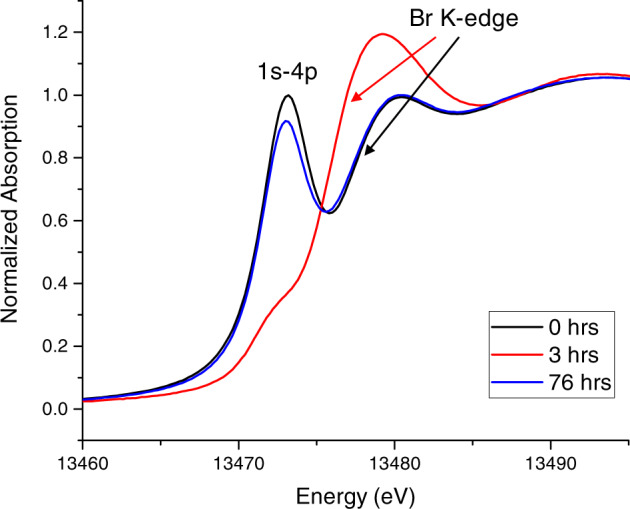
Bromine K-edge XANES on **SFC** exposed to HD simulant. Conditions: 30 mg of SFC exposed to 50 µL of liquid CEES placed directly on the surface of SFC (18 turnovers based on Br_3_^−^) under 1 atm. of air in a closed glass vial at ambient temperature (~22 °C). Aliquots of the slurry mixture were loaded in Kapton capillaries for XANES measurements after 0 h (black), 3 h (red), and 76 h (blue). Arrows indicate the Br K-edges (black arrow corresponds to both blue and black curves).

Further evidence to support the catalytic nature of this material in neat agent/simulant was obtained using bromine X-ray absorption near-edge structure (XANES) and extended X-ray absorption fine structure (EXAFS) spectroscopies. The bromine K-edge XANES shows that throughout the course of the reaction, the initial Br_3_^−^ (characterized by a 1*s*-4*p* pre-edge peak at 13,473 eV)^44–46^ is consumed upon exposure to CEES and then reforms upon reaction completion (Fig. 3) similar to what was observed for the studies done in acetonitrile. The EXAFS spectra (Supplementary Fig. 19) and quantitative analysis (Supplementary Figs. 19 and 20, and Supplementary Table 1) also demonstrate regeneration of the tribromide, as evidenced by the preservation of the average Br–Br lengths of 2.55 ± 0.01 Å and 2.54 ± 0.01 Å before and after CEES exposure, respectively. Finally, copper K-edge EXAFS fitting indicates that, similar to the catalysis in acetonitrile, the coordination environment of the Cu centers switches from O to Br upon exposure to CEES (Supplementary Figs. 21 and 22, and Supplementary Table 2). This is consistent with Cu complexing bromide ions, which aides in the formation of the reactive bromosulfonium intermediate. It also suggests that the role of copper for catalysis in acetonitrile remains the same in neat agent/simulant.

**Fig. 4:**
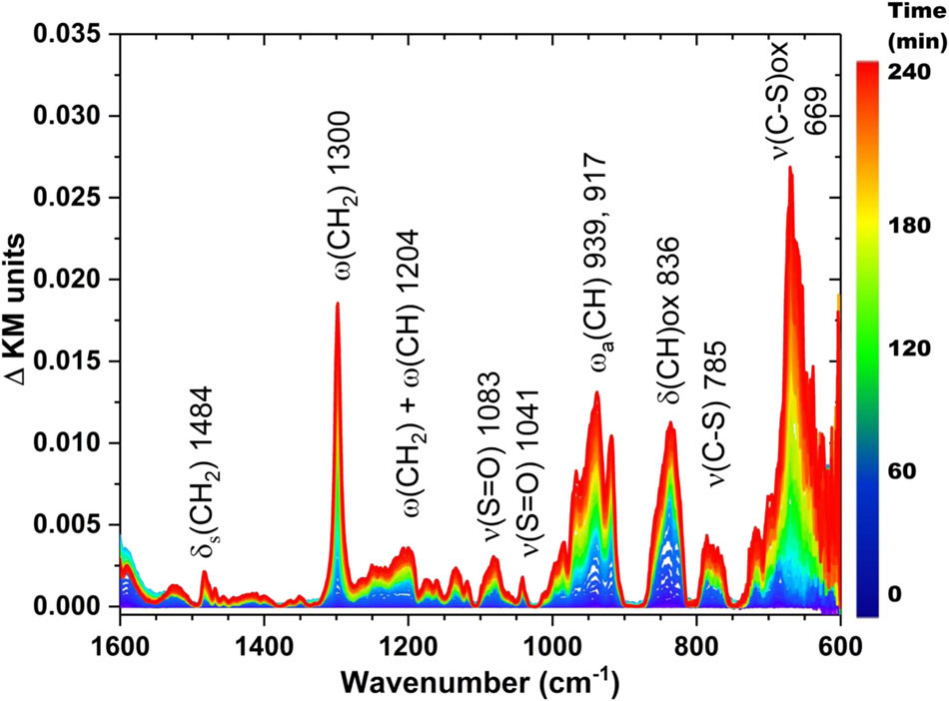
DRIFTS spectra of HD oxidation catalyzed by **SFC**. Conditions: SFC placed in a DRIFTS cup exposed to HD vapor in a gas stream of 2% relative humidity/Zero Air for 4 h.

In addition to exhibiting catalytic turnover with liquid HD/CEES, a significant advantage of this system is that it retains this activity against vapor phase agent/simulant. When SFC is exposed to saturated CEES vapor, there is a loss of the tribromide peak in the Br K-edge XANES spectra (Supplementary Fig. 23) demonstrating reactivity with SFC. For live agent testing, we exposed SFC to a vapor stream of HD, monitoring changes to the material by diffuse reflectance infrared Fourier transform spectroscopy (DRIFTS). As indicated by peaks such as the one at 1300 cm^−1^ corresponding to the HD-based CH_2_ wag, there is an initial physisorption of HD over time. The peaks at 1083 and 1041 cm^−1^, which also increase with time, are consistent with an S=O bond providing evidence for sulfoxide (HDO) product formation (Fig. 4). Immediately following the DRIFTS experiment, the HDO assignment was confirmed by gas chromatography-mass spectrometry (GC-MS) (Supplementary Fig. 24). Thus, the solid catalyst, SFC, exhibits a capacity for both vapor and liquid HD decontamination in the ambient environment.

## Conclusion

In summary, we report the development of a highly reactive solid material for the selective, catalytic, air-based oxidation of HD to decontaminated HDO at ambient conditions. The design of this solvent-free catalyst was informed by mechanistic studies of the homogeneous catalytic system in solution. Cu(II) significantly increases the reaction rate and simultaneously facilitates a colorimetric detection of HD. Several experiments establish that the key intermediate in aerobic sulfoxidation, the bromosulfonium ion, is formed reversibly, and that a central role of Cu(II) in accelerating this process is to increase the concentration of the bromosulfonium during catalytic turnover. These insights resulted in the development of a solid formulation that is active against both liquid and vapor HD. For these solvent-free reactions, the use of XAFS and DRIFTS enabled monitoring of the reaction under turnover conditions along with comparisons to the mechanistic studies conducted in acetonitrile. Collectively, these findings mark substantial progress toward the effective decontamination of HD, as well as our understanding of sulfide oxidation using Br_*y*_/NO_*x*_/O_2_ systems.

## Methods

All chemicals were reagent grade or higher and were used as received unless otherwise specified. CEES, Nafion™, TBABr_3_, and TBANO_3_ were purchased from Sigma-Aldrich and Cu(NO_3_)_2_•3H_2_O was purchased from Sargent-Welch VWR.

All measurements were conducted at ambient temperature (~22 °C).

### Synthesis of 2-chloroethyl ethyl sulfoxide (CEESO) standard

The following synthesis is a modification of previous literature reports.^25,47^ CEES (2 mL) was added dropwise to concentrated nitric acid (10 mL) in a 25 mL round bottom flask with stirring. A reflux condenser was used to minimize the loss of gases from the system, and the round bottom flask was placed in a water bath to maintain a constant temperature as the CEES was added. The solution was stirred for 1 h and then poured over a mixture of ice and water (~50 mL). Once the ice had melted, the product was extracted 3 times with dichloromethane. These dichloromethane layers were combined, and any remaining acid was neutralized twice with 1 M aqueous sodium bicarbonate by shaking the two layers together in a separatory funnel. The dichloromethane layer was then dried with anhydrous magnesium sulfate. The mixture was filtered, and the volume was reduced in a roto-evaporator until only an oil remained, which was the target product, CEESO.

### Gas chromatography measurements

The GC data were collected on an Agilent Hewlett Packard (HP) 6890 GC system with an HP-5 phenyl methyl siloxane column and a flame ionization detector (FID).

### Solution oxidation studies

Stock solutions with concentrations of 50 mM in acetonitrile (MeCN) were prepared for TBABr_3_, TBANO_3_, *p*-TsOH, and Cu(NO_3_)_2_•3H_2_O (in some cases Cu(ClO_4_)_2_•6H_2_O). To prepare catalytic reactions, the necessary volumes of each of these stock solutions were added to a 20-mL glass vial fitted with a polytetrafluoroethylene (PTFE) septum followed by the addition of H_2_O (for experiments including H_2_O) and the 1,3-dichlorobenzene (DCB) internal standard (70 mM). MeCN was added to make the total volume equal to 2 mL after the addition of all components. Solutions were stirred with PTFE-coated stir bars at a rate of 620 rpm. CEES (60 μL, 515 μmol, 103 mM) was added to initiate the reaction. The first time point was taken by GC immediately following the addition of CEES. To keep the partial pressure of O_2_ in the headspace constant, the vials were sealed with PTFE septa after the initial time point and oxygen balloons were attached to the vials. As the reaction proceeds, O_2_ is consumed lowering the pressure in each vial. The O_2_ balloon replaces the lost oxygen thus maintaining constant pressure and composition of the headspace. For each time point, 0.4 μL of the reaction solution was injected on the GC column. Reaction conversion was followed through integration of the CEES peak area relative to that of the 1,3-DCB internal standard. Selectivity was confirmed by ^13^C NMR by comparing to reference standards.

### Solution NMR experiments

Solution ^13^C NMR spectroscopic measurements were made on a Bruker Ascend™ AVANCE III 600 MHz spectrometer with a broadband cryogenic cooler Prodigy CryoProbe™. Additional ^13^C NMR measurements were conducted on a Varian INOVA 400 MHz spectrometer using a Varian DM40P5AP04 probe. All NMR tubes used here have a 5 mm outer diameter (OD) and were spun at 20 Hz.

### Stopped-flow studies

Stock solutions (50 mM) were made for all reagents used in the stopped-flow studies with the exception of CEES and H_2_O. The CEES and H_2_O were added directly to reaction solutions by micropipette. For each experimental solution, reagents were mixed and diluted to the desired concentration in 20 mL scintillation vials capped with PTFE septa. Glass syringes were used to load sample solutions from vials into the instrument. Each sample was passed through the system 7 times to remove all traces of previous experiments. Kinetic curves were obtained using a HI-TECH Scientific KinetAsyst SF-61sX2 sample-handling unit with a diode array spectrophotometer.

### UV-Vis studies

Stock solutions (50 mM) were made for all reagents used in the UV-Vis studies with the exception of CEES and H_2_O. The CEES and H_2_O were added directly to reaction solutions by micropipette. For each experimental solution, reagents were mixed and diluted to the desired concentration in 20 mL scintillation vials capped with PTFE septa. Aliquots of 15 µL from these solutions were diluted 200 times with MeCN creating a 3 mL total volume and transferred to an airtight 1 cm path length quartz cuvette. The dilution allowed accurate measurement of the Br_3_^−^ absorption maximum at 270 nm using an Agilent 8453 UV-visible spectrophotometer. In cases with mixed acetonitrile–water solvent systems, the dilution was done with the same concentration of water to keep this variable constant. In the case where water was removed from the solution, 1.2 g of anhydrous MgSO_4_ was added to the 5 mL solution of 5.0 mM TBABr_3_, 50 mM CEES, and 2% H_2_O following a measurement of the UV spectra. The mixture was vigorously swirled to ensure reaction between the solid MgSO_4_ and H_2_O in the solution. After 15 min of equilibration time, the MgSO_4_ particles were no longer suspended in the solution and a 15 µL aliquot was taken and diluted 200-fold, as described above for the UV measurement.

### Solid-formulation catalyst (SFC) preparation

Nafion™ (150 mg, 136 mmol equivalents of H^+^), TBABr_3_ (145 mg, 300 mmol), TBANO_3_ (61 mg, 200 mmol), and Cu(NO_3_)_2_•3H_2_O (24 mg, 100 mmol) were mechanically mixed with a mortar and pestle. The green solid was then transferred to an airtight container for storage.

### Solid-formulation catalyst (SFC) studies

The SFC (25.3 mg) was added to 5 mL test tubes. CEES (50 μL, 430 μmol) was added directly on top of the solid initiating the reaction. Rubber septa (size 14/20) were used to cap the test tube and O_2_-filled balloons were used to maintain the oxygen concentration in the headspace. A stock solution of 215 mM 1,3-DCB (internal standard) in toluene was made for CEES extraction and quantification from the solid-formulation reactions. The CEES in the test tubes was extracted after a few seconds of shaking the test tube with 2 mL of the 1,3-DCB stock solution and then quantified by GC. CEES and the product CEESO are soluble in toluene while other components are not thus shutting down the catalytic cycle once the toluene is added. As a result, each time point required a separate experiment. Selectivity was confirmed by ^13^C NMR. Coaxial inserts (2 mm stem OD) with D_2_O were used for the lock to avoid introducing additional carbon peaks or the excessive use of deuterated toluene.

### CEES oxidation with SFC under relevant battlefield conditions

#### Hydrocarbons

The SFC (25 mg) was added to 5 mL test tubes. Octane (40 µL, 240 mmol) and CEES (50 µL, 430 µmol) were added directly on top of the solid catalyst. Rubber septa (size 14/20) were used to cap the test tube. Balloons filled with O_2_ were used to maintain the oxygen concentration in the headspace. A stock solution of 215 mM 1,3-DCB (internal standard) in toluene was made for CEES extraction and quantification.

#### Carbon dioxide

The SFC (25 mg) was added to 5 mL test tubes. CEES (50 µL, 430 µmol) was added directly on top of the solid catalyst. Rubber septa (size 14/20) were used to cap the test tube. CO_2_ (150 µL, 6 µmol) was injected through the septa into the test tube using a gastight syringe. CO_2_ was added in a 1:1 molar ratio with Cu(NO_3_)_2_•3H_2_O. Balloons filled with O_2_ were used to maintain the oxygen concentration in the headspace. A stock solution of 215 mM 1,3-DCB (internal standard) in toluene was made for CEES extraction and quantification.

### Live agent studies

#### Liquid HD reaction

The SFC (5 mg) was placed in a sealed vial with a 20 mL syringe filled with O_2_ as the gas headspace. HD (5 μL) was applied to the surface of the SFC. Exposures were run for 1, 2, 4, 8, 24, and 96 h. Exposed powder was placed in a 20 mL scintillation vial and 1.5 mL (reagent grade or better) chloroform was added. The slurry was vortexed for ~60 s and drawn into a 2 mL Luer-slip plastic syringe (National S7510-3) and subsequently filtered through a (0.45 µm × 13 mm diameter) nylon membrane syringe filter into a clear silanized screw-top 2 mL vial (Agilent Technologies, Part no. 5183-2070). This was then placed in the autosampler of an Agilent 6890N Network GC System with a 5973 Network Mass Selective Detector.

#### Vapor HD reaction

The catalyst, SFC, was placed in the DRIFTS cup and equilibrated under a stream of 2% relative humidity/Zero Air in a diffuse reflection accessory (DiffusIR, Pike Technologies, Madison, USA) and a diffuse reflectance cell (DiffusIR environmental chamber, Pike Technologies, Madison, USA). After equilibration, background spectra were collected with 1024 scans per spectra. Data were collected in the following manner: 1024 scans were collected with a collection length of 726.96 s. The instrumental resolution was kept at 2.000 cm^−1^ with levels of zero filling kept at zero. Each scan consisted of 33,056 total points with 32,768 fast Fourier transform (FFT) points. The laser frequency utilized was 15,798.25 cm^−1^, and the interferogram peak position was 16,384 cm^−1^. The apodization was Happ-Genzel and the phase correction was Mertz. The data represent 3735 points between 599.7627 cm^−1^ and 4200.2676 cm^−1^ with a spectral interval after FFT of 0.964249 cm^−1^. The spectrometer is a Nicolet 6700 (Nicolet, Thermo Fisher Scientific, Waltham, MA), with an IR source, MCT/A detector and a KBr beam splitter. The optical velocity is 3.7974 cm/s with an aperture of 65.00% and a sample gain of 2.0. The high-pass filter is set to 200.0000 Hz and the low-pass filter is set to 50,000.0000 Hz. After the background spectrum was collected, HD was introduced from a micro-fritted glass saturator (Glassblowers, Inc., NJ) that holds the HD liquid sits in an isothermal water bath held at 20 °C, into the humid Zero Air Stream, and then difference spectra were collected for 4 h.

### X-ray absorption fine structure (XAFS) experiments

XAFS experiments were performed at National Synchrotron Light Source (NSLS) II, Beamline 7-BM quick X-ray absorption and scattering (QAS). For the solid–liquid CEES exposure (Fig. 3), 30 mg of SFC was exposed to 50 µL of liquid CEES under 1 atm. of air in a closed glass vial for 0, 3, and 76 h. Aliquots of the slurry mixture were loaded in Kapton capillaries for XAFS measurements. For the in situ solid–gaseous CEES exposure (Supplementary Fig. 16), 4 mg of SFC was loaded in a Kapton capillary with both ends open and the capillary was fixed inside a Nashner-Adler cell, a sealed jar customized for XAFS measurements. Liquid CEES (2 mL) was soaked in the cotton wool placed in a beaker with the volume of 3 mL at the bottom of the cell and produced CEES vapors. The cell was sealed under ambient air. Br K-edge (~13,478 eV) XAFS data, collected in transmission mode with 45 s per spectrum, were simultaneously measured with Au foil with the L_2_-edge at 13,734 eV for energy alignment and calibration purposes. Cu K-edge (~8979 eV) XAFS data, collected in fluorescence mode with 45 s per spectrum, were simultaneously measured with Cu foil for energy alignment and calibration purposes. XAFS data were processed and analyzed using Athena and Artemis^48^ programs of the IFEFFIT package^49^. Quantitative analysis of Br K-edge EXAFS was performed by fitting theoretical EXAFS spectra to the experimental data in *r*-space. The fitting model was constructed by adopting a structure of Br_2_ and the scattering contribution from a Br–Br bond of 2.301 Å was included. The amplitude factor was fixed to be 0.80. Quantitative analysis of Cu K-edge EXAFS was performed by fitting theoretical EXAFS spectra to the experimental data in *r*-space. The fitting model for Cu K-edge EXAFS of SFC before CEES exposure was constructed by adopting a structure of Cu(NO_3_)_2_ and the scattering contribution from a Cu–O bond of 1.959 Å was included. The fitting model for Cu K-edge EXAFS of SFC after CEES exposure for 3 h was constructed by adopting a structure of CuBr_2_ and CuS, and the scattering contribution from a Cu–Br bond of 2.420 Å and a Cu–S bond of 2.353 Å were included. The amplitude factor was fixed to be 0.85.

## Supplementary information

Supplementary information

## Data Availability

All data relating to the findings of this study are available in the Supplementary information or available from the corresponding author upon reasonable request.
