# Supplementary material for: A solvent-free solid catalyst for the selective and color-indicating ambient-air removal of sulfur mustard
Source: Commun Chem. 2021 Mar 8;4:33. doi: 10.1038/s42004-021-00465-7 (PMC9814880; doi:10.1038/s42004-021-00465-7)
Supplement: Supplementary file 1 — Supplementary information [file 42004_2021_465_MOESM1_ESM.pdf]

Supporting Information

A Solvent-free, Solid Catalyst for the Fast, Selective, and Color Indicating Ambient-Air Removal of Mustard

Daniel L. Collins-Wildman[1], Kevin P. Sullivan[1], Yurii V. Geletii[1], Victoria G. Snider[1], Wesley O. Gordon[2], Alex Balboa[2], Yiyao Tian[3], Rachel M. Slaugenhaupt[1], Alexey L. Kaledin[4], Christopher J. Karwacki[2], Anatoly I. Frenkel[3,5], Djamaladdin G. Musaev[1,4], Craig L. Hill[*,1]

[1]Department of Chemistry, Emory University, Atlanta, GA 30322, United States
[2]U.S. Army Combat Capabilities Development Command Chemical Biological Center, Aberdeen, Maryland 21010, United States
[3]Department of Materials Science and Chemical Engineering, Stony Brook University, Stony Brook, New York 11794, United States
[4]Cherry L. Emerson Center for Scientific Computation, Emory University, Atlanta, Georgia 30322, United States
[5]Chemistry Division, Brookhaven National Laboratory, Upton, New York 11973, United States
[*]Corresponding Author
chill@emory.edu

Table of Contents:

Supplementary Figs. 1-24 (p. 2-24)

Supplementary Tables 1 and 2 (p. 19, 22)

List of Abbreviations (p. 24-25)

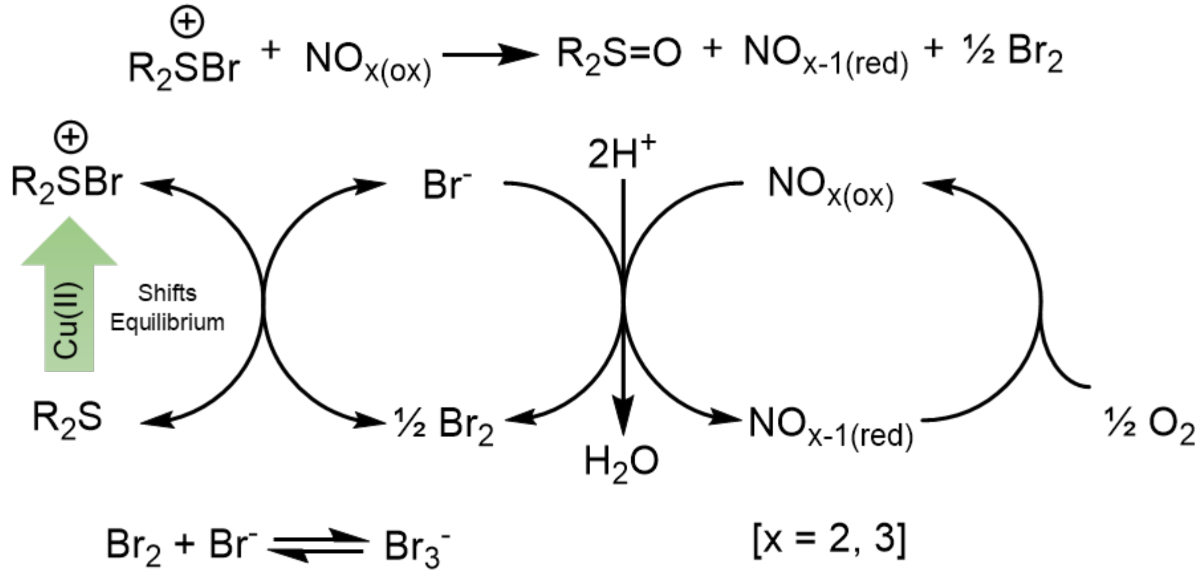

Supplementary Figure 1. Proposed general reaction scheme for oxygen-based catalytic and selective oxidation of sulfides (R_2S), such as HD, to sulfoxides using Br_3^- and NO_3^- salts. The green arrow indicates how the presence of Cu(II) shifts the reversible reaction of R_2S with Br_2 to form R_2SBr^+ and Br^- in favor of the products.

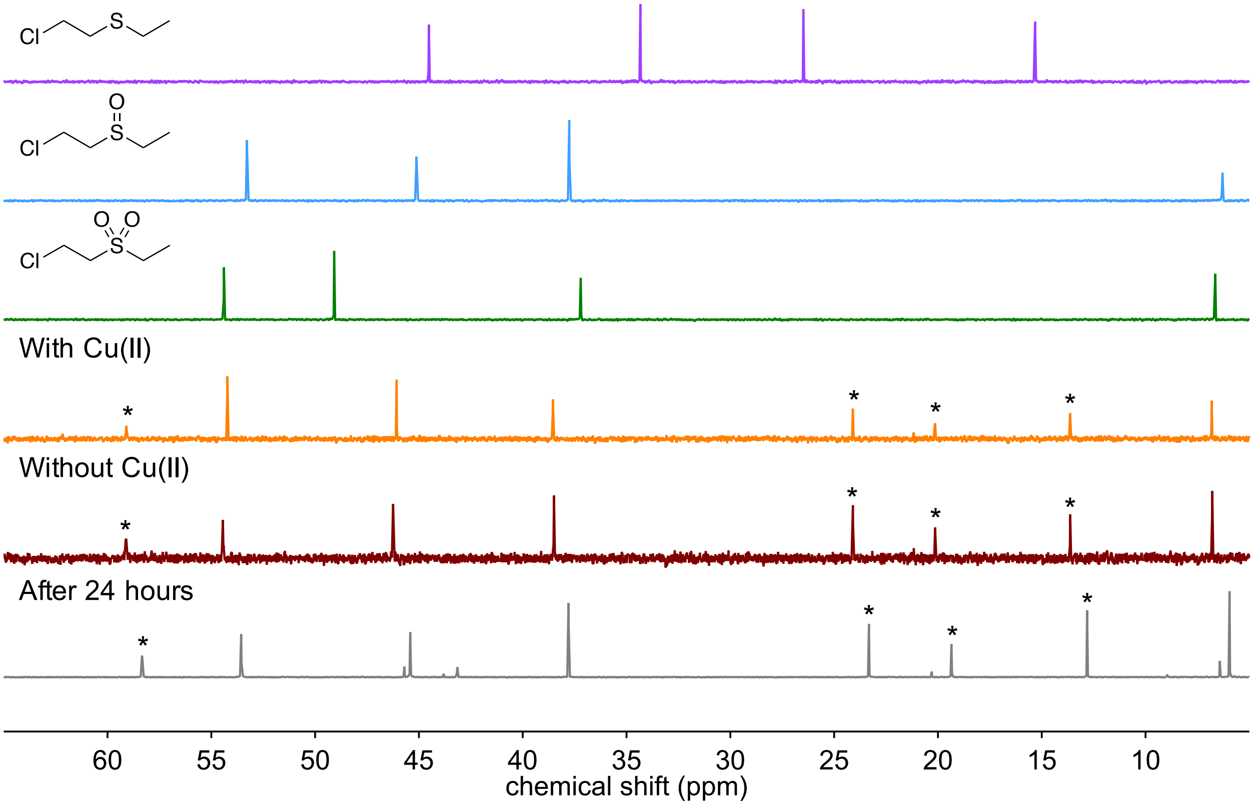

Supplementary Figure 2. ^{13}C NMR of sulfide standards (purple, blue, and green curves) and CEES reaction products (orange, brown, and grey curves). Conditions for catalytic trials prior to NMR analysis: 100 mM CEES, 5.0 mM $TBANO_3$, 5.0 mM $TBABr_3$, 10 mM *p*-TsOH, 2% H_2O and 10% CD_3CN (v/v) in MeCN at ambient temperature (~22 ºC) under 1 atm of air. Reaction run with copper: $[Cu(ClO_4)_2 \cdot 6H_2O]$ = 1.5 mM. (*) = TBA counter cations. $CEESO_2$, CEESO and CEES standards were dissolved in CD_3CN (small differences between standard and reaction solution compositions result in slightly different chemical shifts). For the grey curve, the spectrum was taken 24 h after reaction completion indicating complete oxidative selectivity for the sulfoxide.

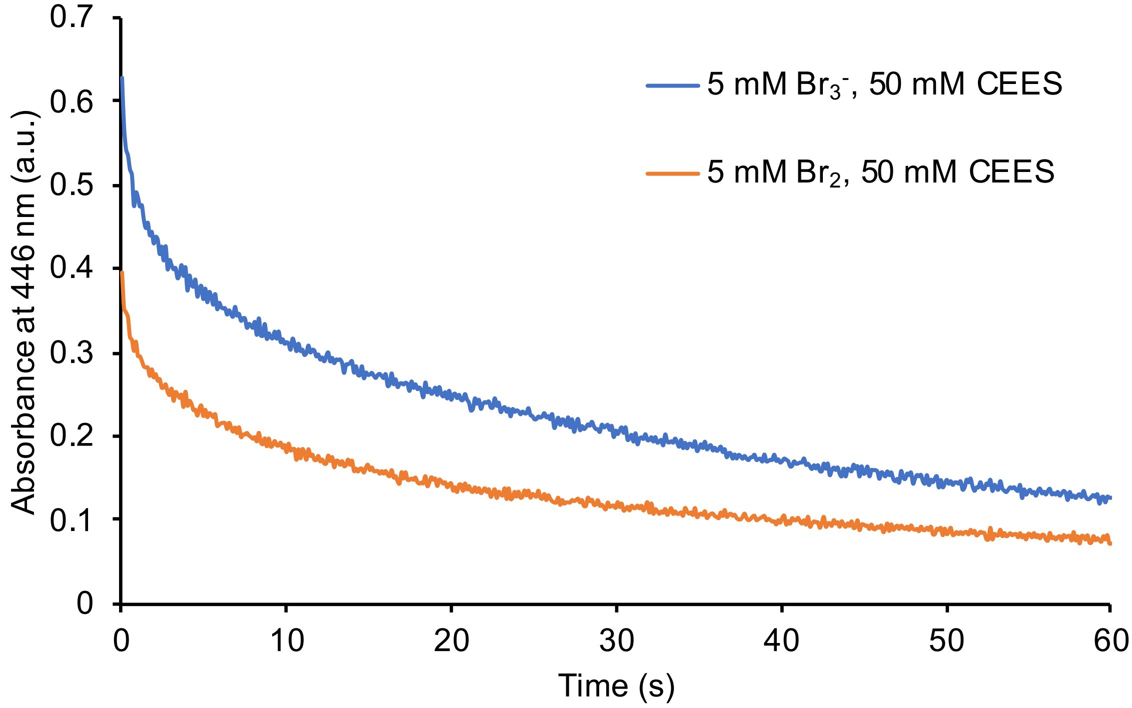

Supplementary Figure 3. Stopped-flow kinetics of 5.0 mM Br_2 (orange) or 5.0 mM $TBABr_3$ (blue) consumption in the reaction with 50 mM CEES measured by the decrease of absorbance at 446 nm. Conditions: 2% H_2O (v/v), 17 mM $HClO_4$, in MeCN at ambient temperature (~22 ºC).

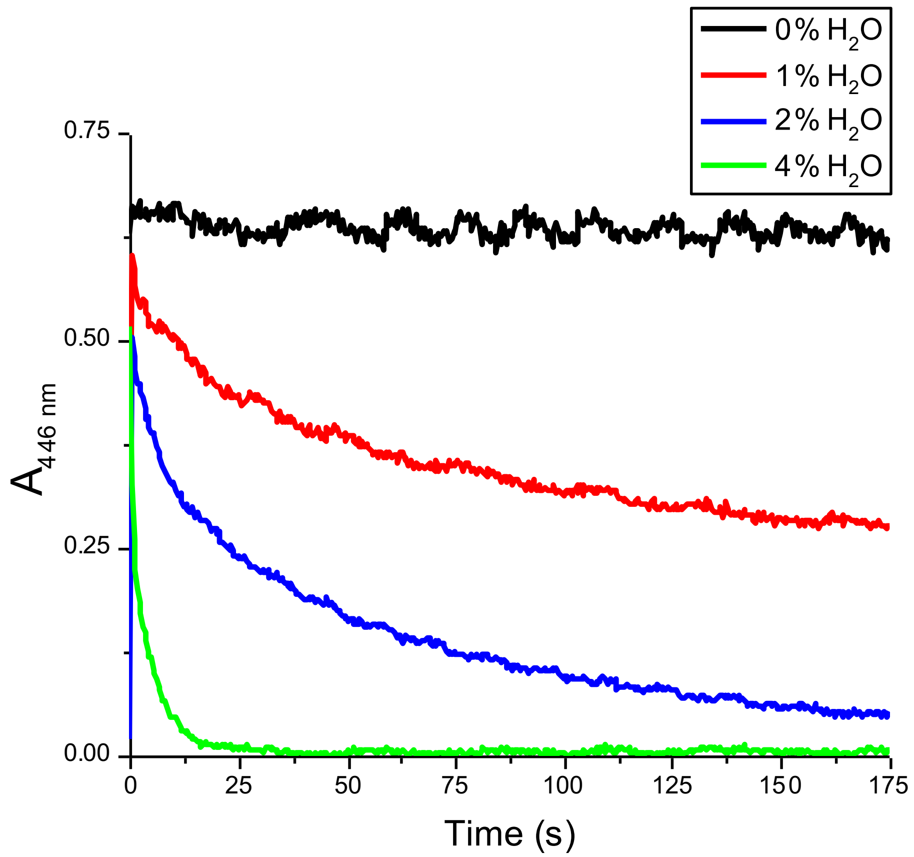

Supplementary Figure 4. Stopped-flow kinetics of CEES reactivity with Br_2/Br_3^- as a function of water content. The reaction is followed by the decrease of the Br_2/Br_3^- isosbestic absorption at 446 nm. Conditions: 5.0 mM $TBABr_3$, 17 mM $HClO_4$, 50 mM CEES, in MeCN at ambient temperature (~22 ºC). Varied water percentage (v/v): 0% (black), 1% (red), 2% (blue), 4% (green).

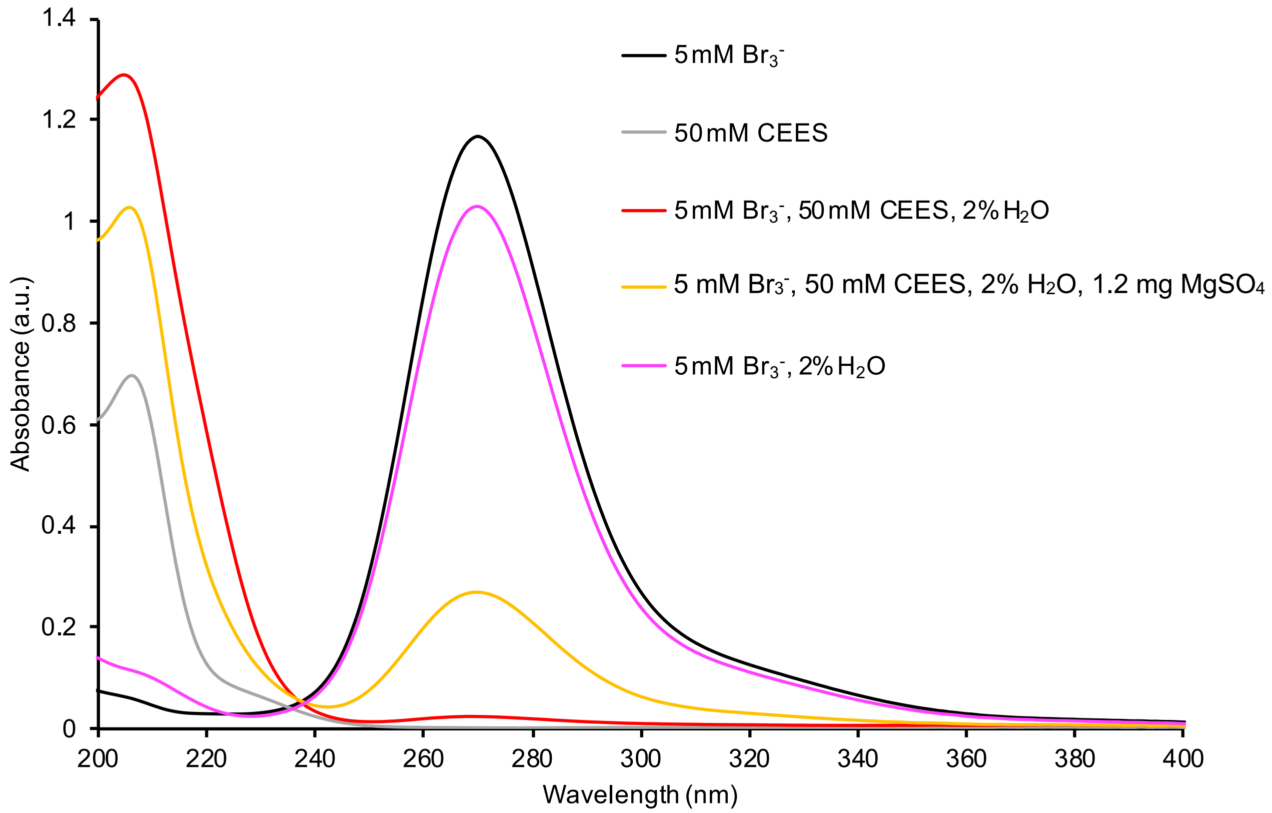

Supplementary Figure 5. UV spectra of Br_3^- absorption (lambda max = 270 nm) with varied water and CEES concentrations. Samples were prepared at stated concentrations at ambient temperature (~22 ºC) in MeCN (5 mL total volume) and then diluted 200-fold to measure the UV spectra. Black curve = 5 mM $TBABr_3$; grey curve = 50 mM CEES; yellow curve = 5 mM $TBABr_3$, 50 mM CEES, 2% H_2O; red curve = 5 mM $TBABr_3$, 50 mM CEES, 2% H_2O, 1.2 g $MgSO_4$; purple curve = 5 mM $TBABr_3$, 2% H_2O. For the samples containing both CEES and Br_3^- (red and yellow traces), the solution was allowed to equilibrate for 15 minutes before measurement and another 15 minutes after the addition of $MgSO_4$.

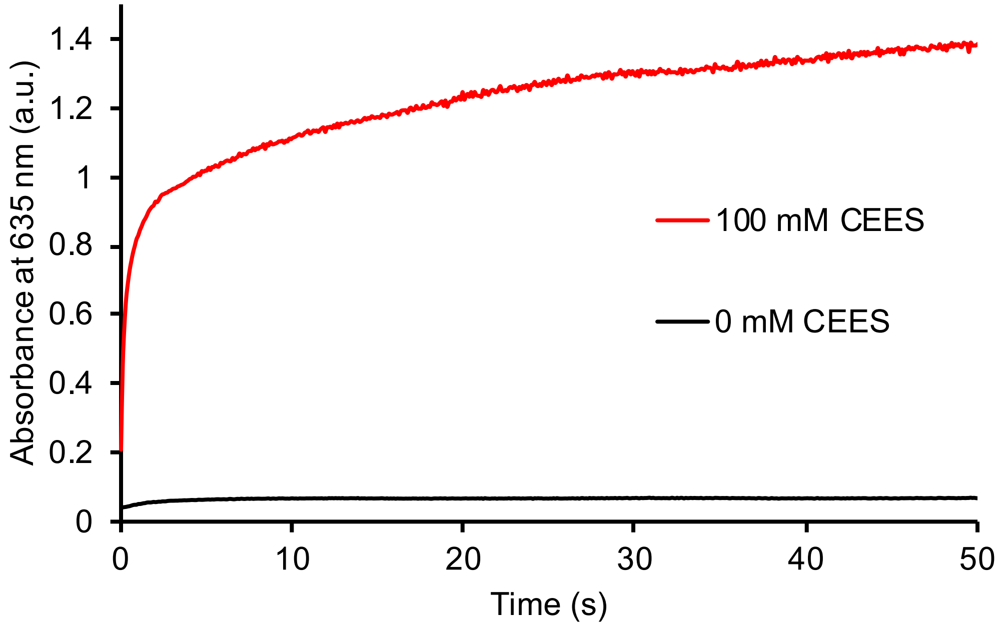

Supplementary Figure 6. Stopped-flow kinetics of $CuBr_3^-$ formation in the presence and absence of CEES. The reaction is followed by the growth of $CuBr_3^-$ absorption at 635 nm. Conditions: 5 mM $TBABr_3$, 1 mM $Cu(ClO_4)_2$•$6H_2O$, in MeCN at ambient temperature (~22 ºC). Red curve = 103 mM CEES, black curve = 0 mM CEES.

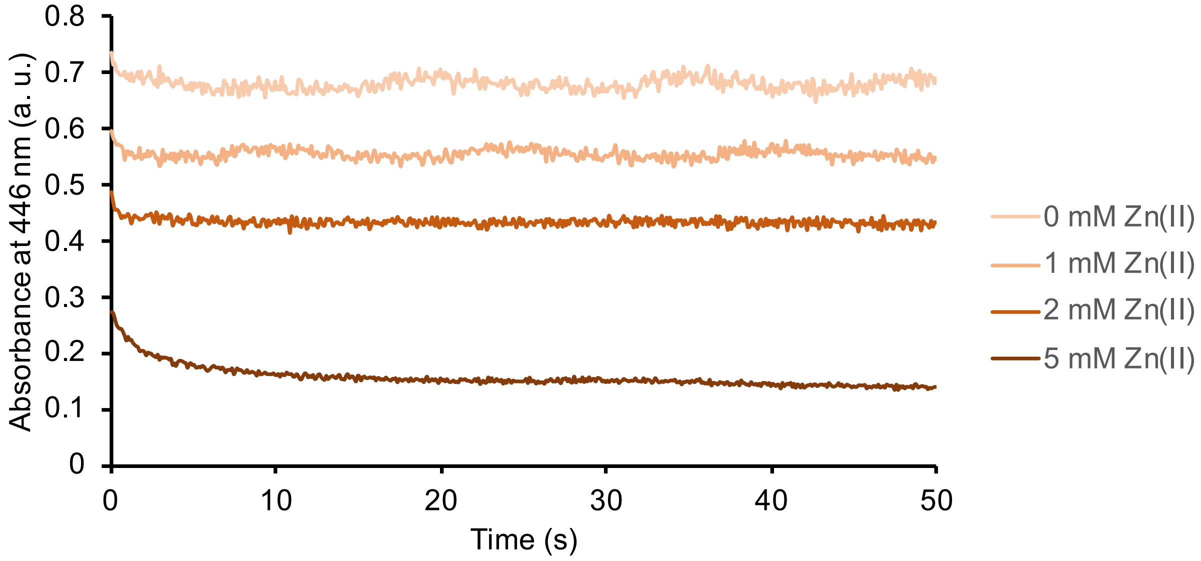

Supplementary Figure 7. Stopped-flow kinetics of CEES reactivity with Br_2/Br_3^- as a function of $[Zn(BF_4)_2]$. The reaction is followed by the decrease of the Br_2/Br_3^- isosbestic absorption at 446 nm. Conditions: 5.0 mM $TBABr_3$, 103 mM CEES, in MeCN at ambient temperature (~22 ºC). Varied zinc concentration: 0 mM to 5 mM $Zn(BF_4)_2$ (light to dark maroon respectively).

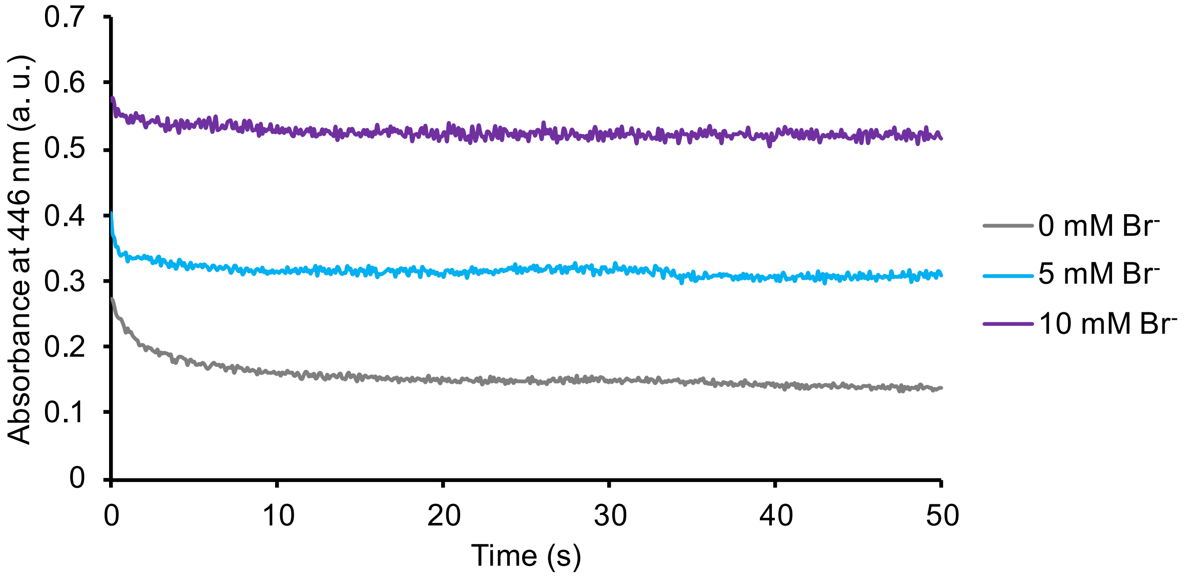

Supplementary Figure 8. Stopped-flow kinetics of CEES reactivity with Br_2/Br_3^-. The reaction is followed by the decrease of the Br_2/Br_3^- isosbestic absorption at 446 nm. Conditions: 5 mM $TBABr_3$, 103 mM CEES, 10 mM $Zn(BF_4)_2$, in MeCN at ambient temperature (~22 ºC), varied concentrations of TBABr: 0 mM (grey), 5 mM (blue), 10 mM (purple).

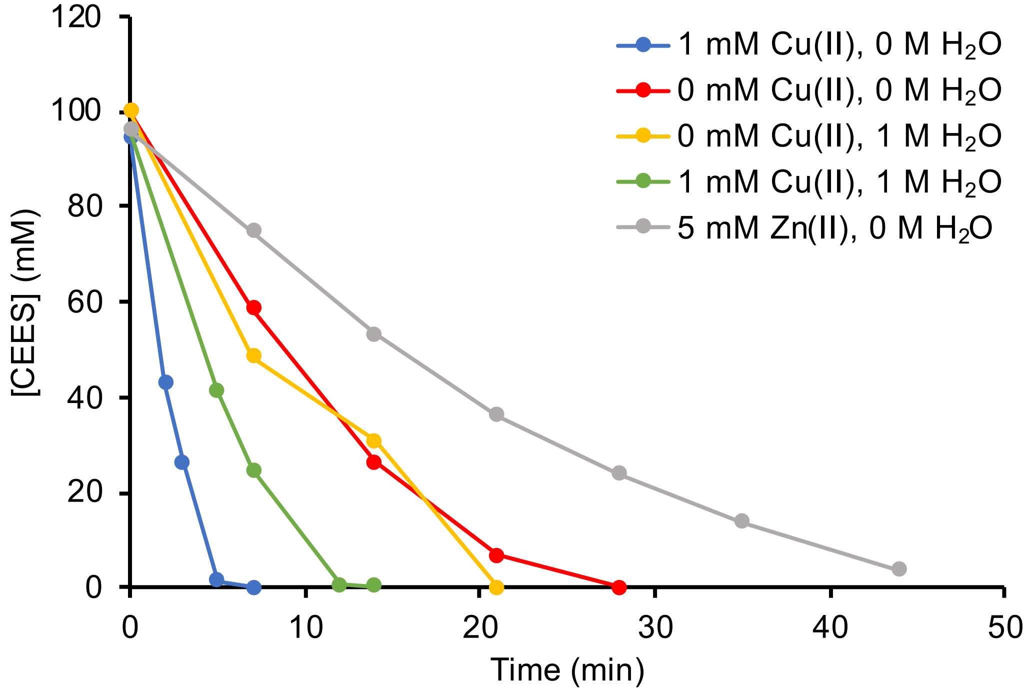

Supplementary Figure 9. Kinetics of CEES oxidation under full catalytic conditions with and without Cu(II), H_2O, and Zn(II). Conditions: 5 mM $TBABr_3$, 10 mM *p*-TsOH, 10 mM $TBANO_3$, 103 mM CEES, 70 mM 1,3-dichlorobenzene (1,3-DCB) internal standard, in MeCN at ambient temperature (~22 ºC), under 1 atm of air. Blue curve = 1 mM $Cu(ClO_4)_2$, 0 M H_2O; red curve = 0 mM $Cu(ClO_4)_2$, 0 M H_2O; orange curve = 0 mM $Cu(ClO_4)_2$, 1 M H_2O; green curve = 1 mM $Cu(ClO_4)_2$, 1 M H_2O; grey curve = 5 mM $Zn(BF_4)_2$, 0 M H_2O. These curves represent 10 turnovers based on NO_3^-.

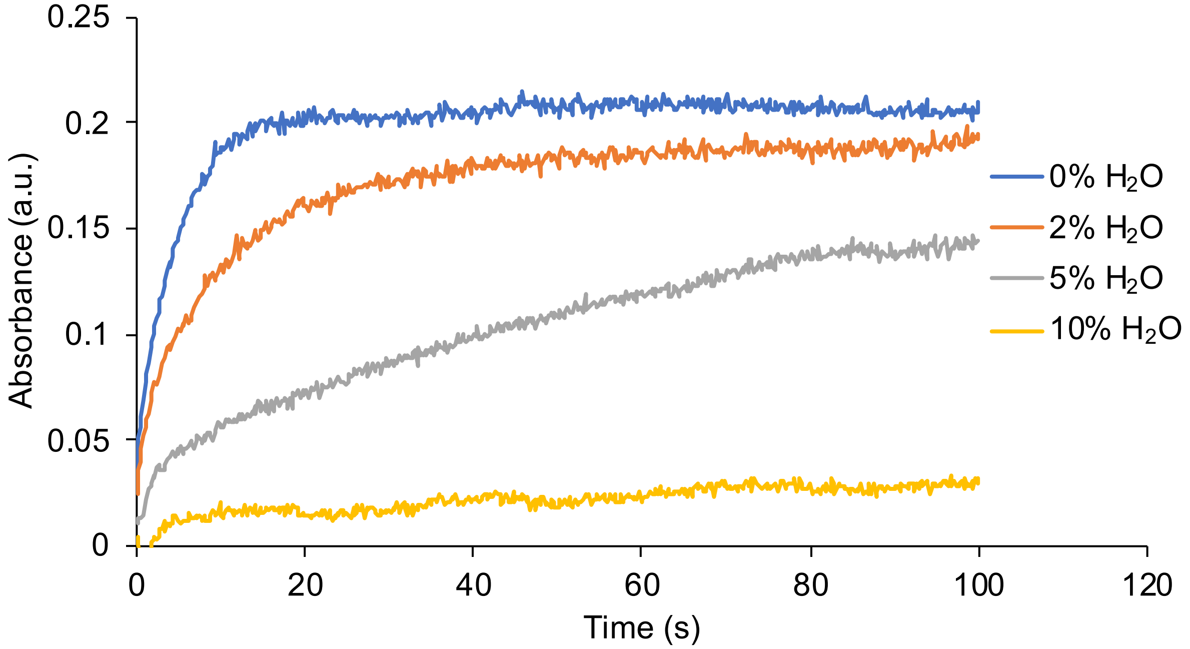

Supplementary Figure 10. Stopped-flow kinetics of Br^- oxidation to form Br_2/Br_3^-. The reaction is followed by the increase of the Br_2/Br_3^- isosbestic absorption at 446 nm. Conditions: 15 mM TBABr, 5 mM $TBANO_2$, 5 mM *p*-TsOH, in MeCN at ambient temperature (~22 ºC). Varied water percentage (v/v): 0% (blue), 2% (orange), 5% (grey), 10% (yellow).

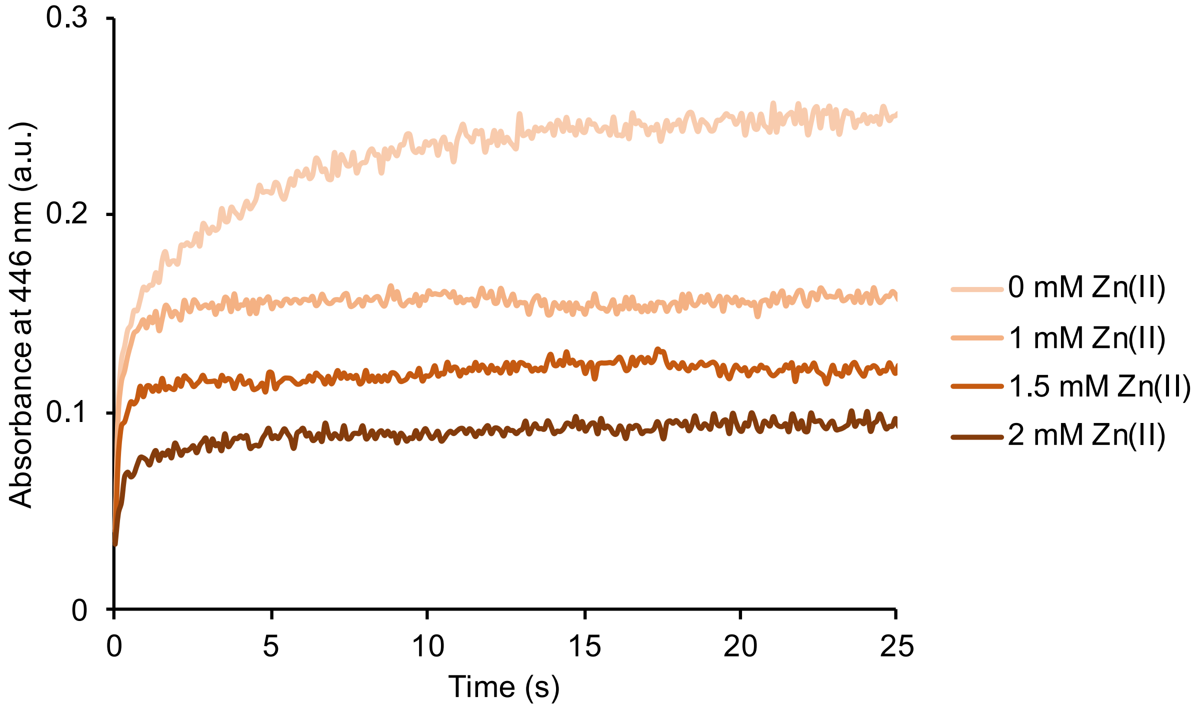

Supplementary Figure 11. Stopped-flow UV-vis of Br^- oxidation to form Br_2/Br_3^-. Syringe 1: 20 mM *p*-TsOH, 10 mM TBA-NO_2 in acetonitrile (MeCN). Syringe 2: 10 mM TBA-Br, varied concentrations of $Zn(BF_4)_2$. Absorbance measurements taken at 446 nm.

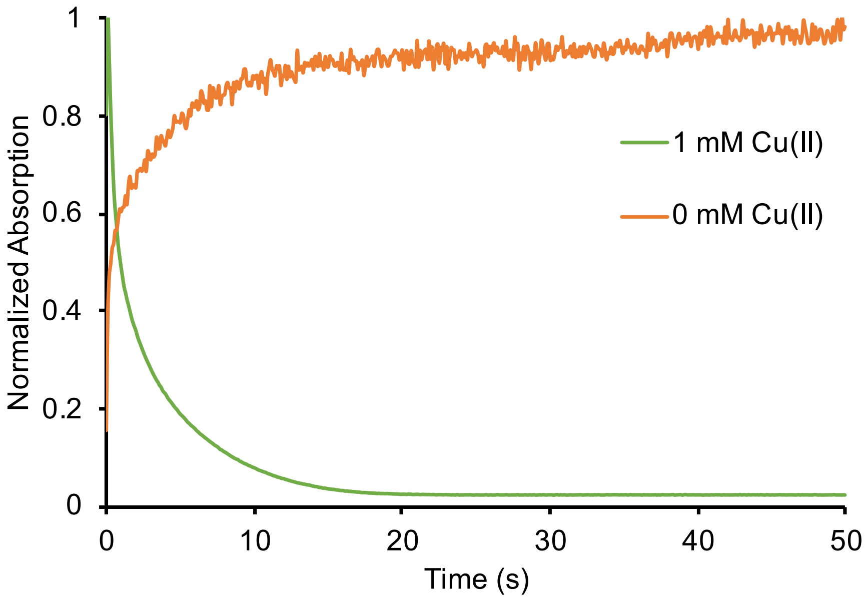

Supplementary Figure 12. Stopped-flow kinetics of Br^- oxidation to form Br_2/Br_3^-. The reaction is followed by the increase of the Br_2/Br_3^- isosbestic absorption at 446 nm (orange) and the decrease of $CuBr_3^-$ absorption at 635 nm (green). Conditions: 5 mM TBABr, 5 mM $TBANO_2$, 10 mM *p*-TsOH, in MeCN at ambient temperature (~22 ºC). Green curve = 1 mM $Cu(ClO_4)_2$•$6H_2O$; orange curve = 0 mM $Cu(ClO_4)_2$•$6H_2O$. Absorbances were normalized for ease of comparison.

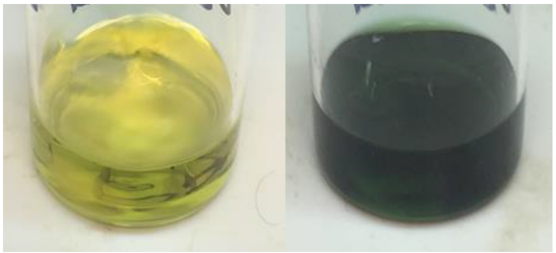

Supplementary Figure 13. Responsive color change behavior in the homogeneous Br_x/NO_x/Cu(II) system. Left: solution containing 5 mM $TBABr_3$, 5 mM $TBANO_3$, 1.7 mM $Cu(ClO_4)_2$•$6H_2O$, 10 mM *p*-TsOH, and 2% H_2O (v/v) in MeCN. Right: the same system

immediately after addition of 100 mM CEES. Solutions were at ambient temperature (22±1 ºC) under 1 atm of air.

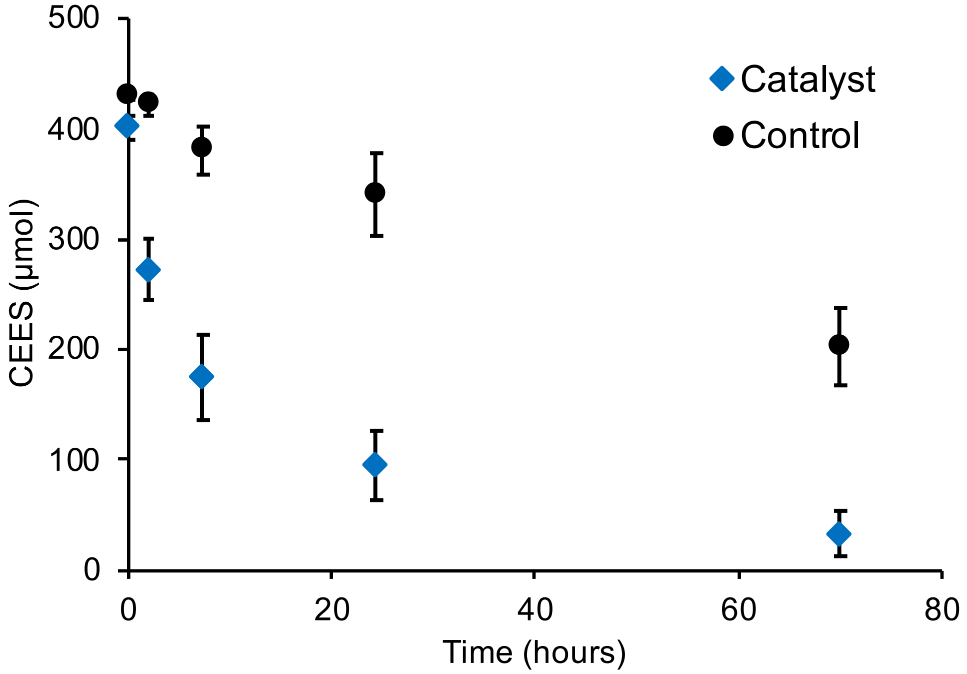

Supplementary Figure 14. Kinetics of CEES oxidation catalyzed by **SFC**. Conditions: 50 µL (430 µmol) of neat CEES added directly to 25.3 mg of the **SFC** (9.6 mg $TBABr_3$, 4.1 mg $TBANO_3$, 1.6 mg $Cu(NO_3)_2$•$3H_2O$, 10 mg Nafion™), under 1 atm of air at ambient temperature (~22 ºC). The control was done in the absence of **SFC**. For the control, CEES concentration falls as a result of evaporation, not oxidation. Measurements taken by GC in triplicate with standard deviation error shown.

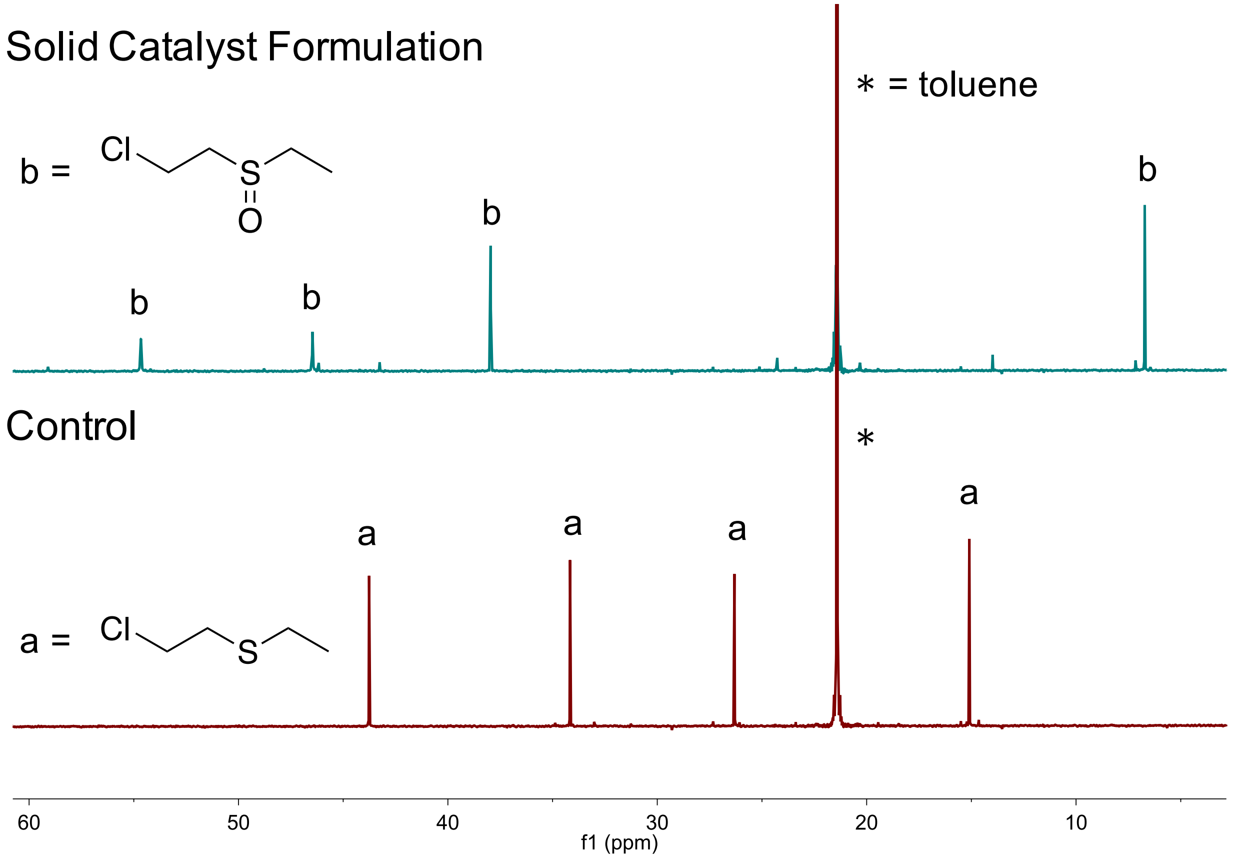

Supplementary Figure 15. ^{13}C NMR of products of neat CEES reactions extracted into toluene (see materials and methods). Conditions for catalytic trials prior to NMR analysis: 50 μL (430 μmol) of neat CEES, 25.3 mg of **SFC** = 9.6 mg $TBABr_3$, 4.1 mg $TBANO_3$, 1.6 mg $Cu(NO_3)_2$•$3H_2O$, 10 mg Nafion™, under 1 atm of air at ambient temperature (~22 ºC). Coaxial inserts filled with D_2O were used to generate the lock signal.

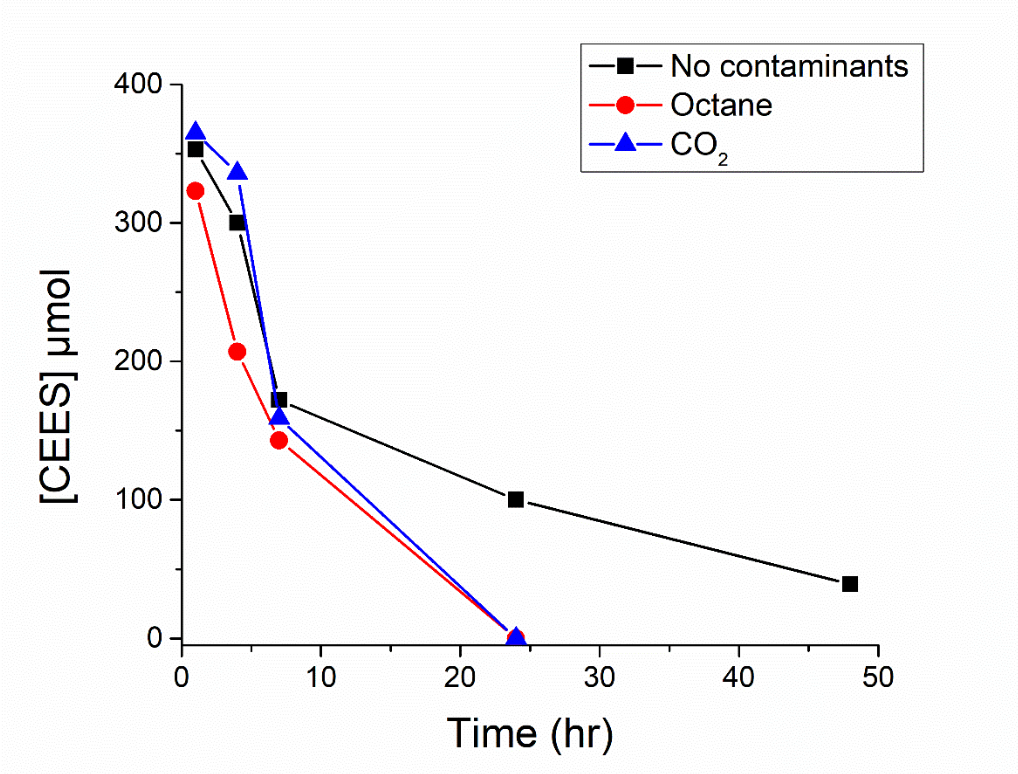

Supplementary Figure 16. Kinetics of CEES oxidation with and without common battlefield contaminants. Conditions: 50 µL (430 µmol) of neat CEES was added directly to 25 mg of SFC (9.6 $TBABr_3$, 4.1 mg $TBANO_3$, 1.6 mg $Cu(NO_3)_2 \cdot 2H_2O$, 10 mg Nafion™), under 1 atm of air at ambient temperature (~22 ºC). (Black) control with no battlefield contaminants; (red) in the presence of hydrocarbons, namely 40 µL (240 µmol) octane; and (blue) with 150 µL (6 µmol) CO_2 in a 1:1 ratio with Cu(II).

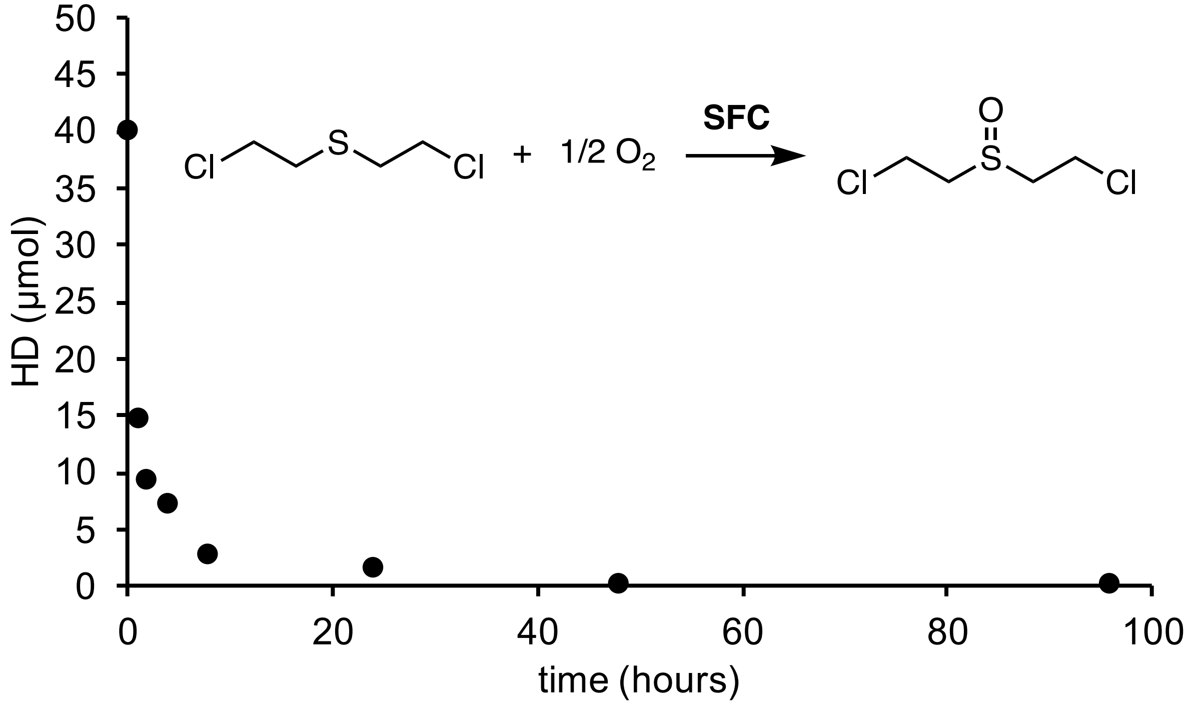

Supplementary Figure 17. Kinetics of HD oxidation catalyzed by **SFC**. Conditions: 5 µL HD added directly to 5 mg of **SFC** at ambient temperature (~22 ºC). Reaction conducted in sealed vial with a 20 mL syringe filled with O_2 as the headspace gas. GC-MS measurements were taken via 1.5 mL $CDCl_3$ extraction.

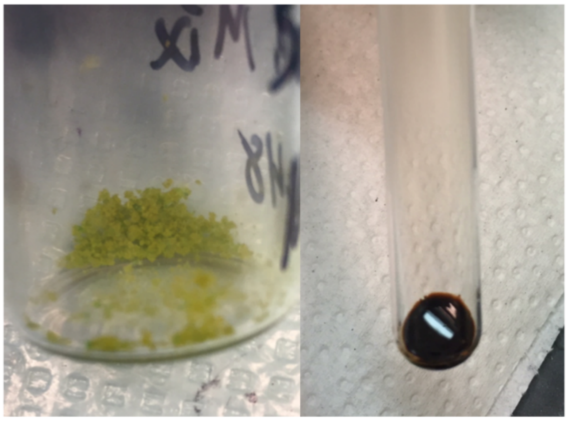

Supplementary Figure 18. Responsive color change behavior in **SFC**. Left: **SFC** with $Br_x/NO_x/Cu(II)/$ Nafion™ ratios of 5.0:3.3:1.7:2.3 respectively. Right: 25.3 mg of **SFC** exposed to 50 μL (430 μmol) of neat CEES.

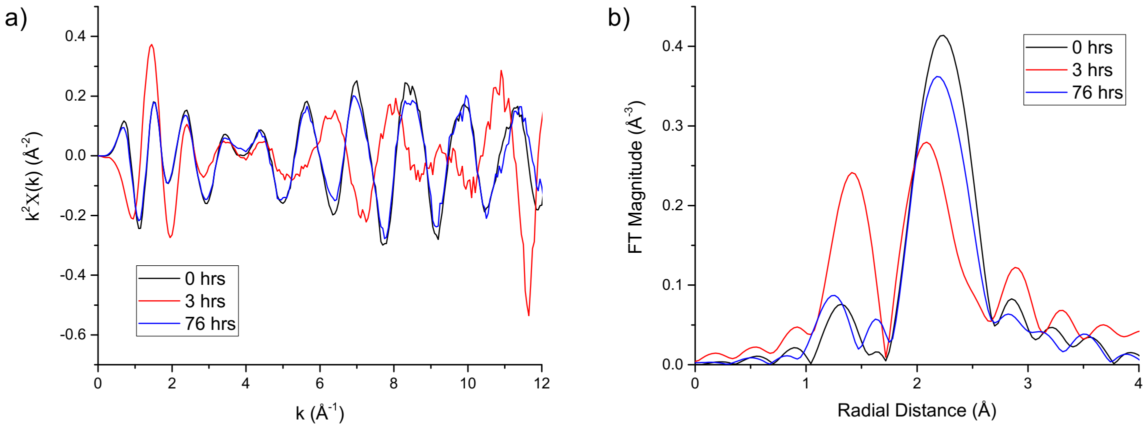

Supplementary Figure 19. Bromine K-edge EXAFS on 30 mg of **SFC** exposed to 50 µL of liquid CEES under 1 atm of air in a closed glass vial at ambient temperature (~22 ºC). Aliquots of the slurry mixture were loaded in Kapton capillaries for EXAFS measurements. Plots of both k-space (a) and r-space (b) data after 0 (black), 3 (red), and 76 (blue) hours. The r-space data was generated with a k-range from 3 $Å^{-1}$ to 12 $Å^{-1}$, with k^2-weighting in Fourier transforms.

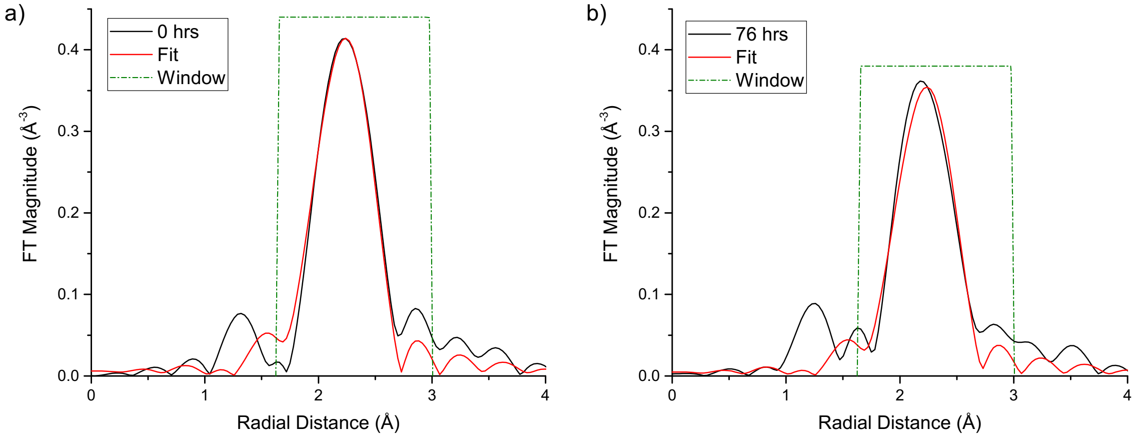

Supplementary Figure 20. Fitting of bromine K-edge EXAFS data on 30 mg of **SFC** exposed to 50 µL of liquid CEES under 1 atm of air at ambient temperature (~22 ºC) in a closed glass vial. Aliquots of **SFC** were loaded in Kapton capillaries for EXAFS measurements both before CEES exposure (a) and after 76 hours (b).

	**0-hour**	**76-hour**
ΔE (eV)	9 ± 2	
N	1.3 ± 0.2	1.0 ± 0.2
R (Å)	2.55 ± 0.01	2.54 ± 0.01
σ^2 (Å^2)	0.006 ± 0.001	0.005 ± 0.002

Supplementary Table 1. Values for Br-Br_N coordination number (N), effective Br-Br interatomic distance (R), and mean squared bond length disorder (σ^2) based on analysis of bromine K-edge EXAFS on 30 mg of **SFC** exposed to 50 µL of liquid CEES under 1 atm of air at ambient temperature (~22 ºC) in a closed glass vial. The fitting was performed in r-space,

using the k-range from 3 Å^{-1} to 12 Å^{-1}, with k^2-weighting in Fourier transforms. Aliquots of **SFC** were loaded in Kapton capillaries for EXAFS measurements both before CEES exposure and after 76 hours. The energy origin correction, ΔE, was constrained in the fit to be the same for the two data sets.

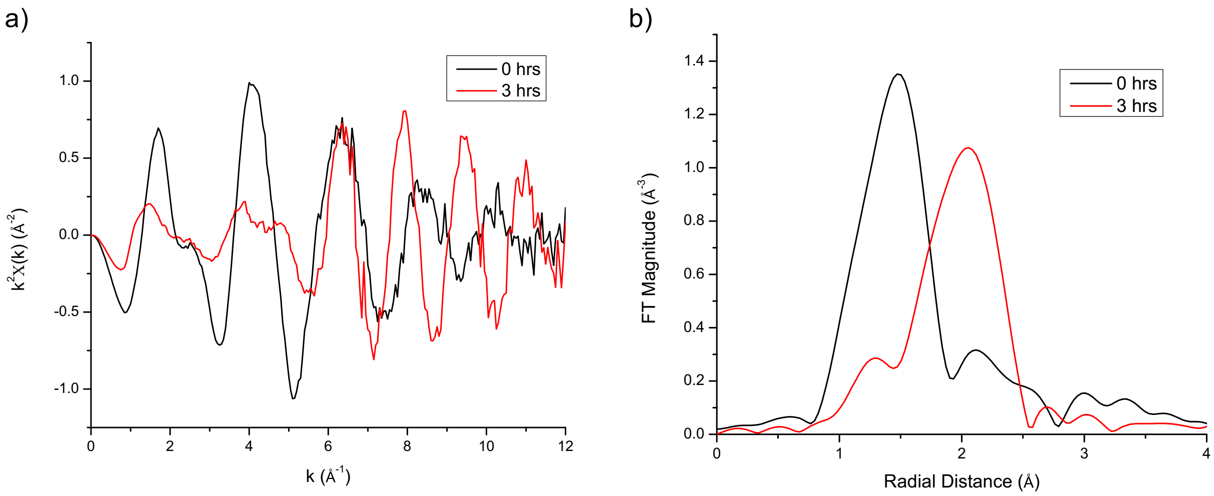

Supplementary Figure 21. Copper K-edge EXAFS on 30 mg of **SFC** exposed to 50 µL of liquid CEES under 1 atm of air in a closed glass vial at ambient temperature (~22 ºC). Aliquots of the slurry mixture were loaded in Kapton capillaries for EXAFS measurements. Plots of both k-space (a) and r-space (b) data after 0 (black) and 3 (red) hours. The r-space data was generated with a k-range from 2 Å^{-1} to 12 Å^{-1}, with k^2-weighting in Fourier transforms.

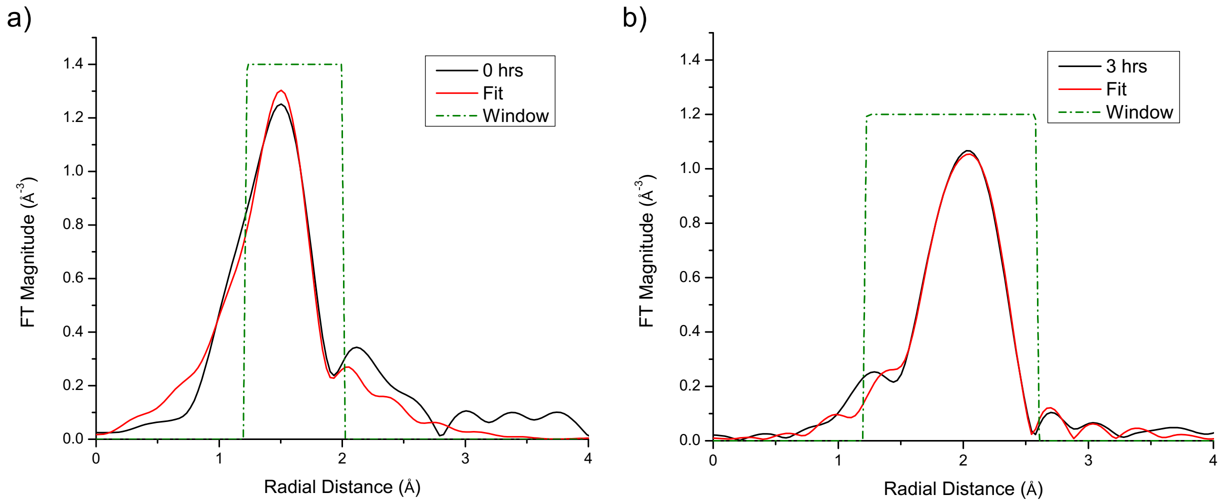

Supplementary Figure 22. Fitting of copper K-edge EXAFS data on 30 mg of **SFC** exposed to 50 µL of liquid CEES under 1 atm of air in a closed glass vial at ambient temperature (~22 ºC). Aliquots of **SFC** were loaded in Kapton capillaries for EXAFS measurements both before CEES exposure (a) and after 3 hours (b).

	0-hour	3-hour
N_O	4.8 ± 0.6	-
R_O (Å)	1.95 ± 0.01	-
σ^2_O (Å^2)	0.006 ± 0.001	-
N_{Br}	-	3.3 ± 0.7
R_{Br} (Å)	-	2.37 ± 0.01
σ^2_{Br} (Å^2)	-	0.007 ± 0.001
N_S	-	1.0 ± 0.4
R_S (Å)	-	2.33 ± 0.03
σ^2_S (Å^2)	-	0.007 ± 0.001

Supplementary Table 2. Values for coordination number (N), effective interatomic distance (R), and mean squared bond length disorder (σ^2) for Cu-O/Br/S bonds based on analysis of copper K-edge EXAFS on 30 mg of **SFC** exposed to 50 µL of liquid CEES under 1 atm of air in a closed glass vial at ambient temperature (~22 ºC). The fitting was performed in r-space, using the k-range from 3 Å^{-1} to 12 Å^{-1}, with k^2-weighting in Fourier transforms. Aliquots of **SFC** were loaded in Kapton capillaries for EXAFS measurements both before CEES exposure and after 3 hours. The energy origin correction, ΔE, for Cu-O and Cu-Br/S bonds were different variables, which were 4.1 ± 1.5 and 0.2 ± 1.1, respectively. σ^2 was set to be same for Cu-Br and Cu-S bonds.

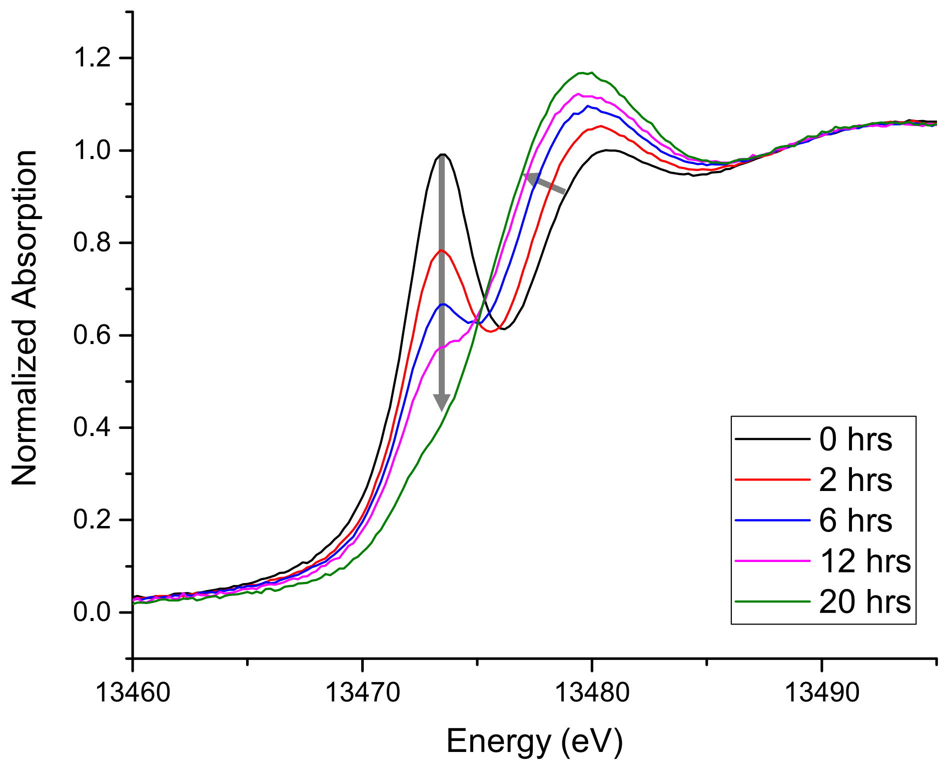

Supplementary Figure 23. Bromine K-edge XANES of **SFC** exposed to saturated CEES vapor under 1 atm of air at ambient temperature (~22 ºC) in a sealed jar. Measurements were taken at different time intervals following initial CEES exposure. The solid catalyst, **SFC**, was packed in Kapton capillaries (ID: 0.11049 cm). Grey arrows indicate the direction of change as a function of time.

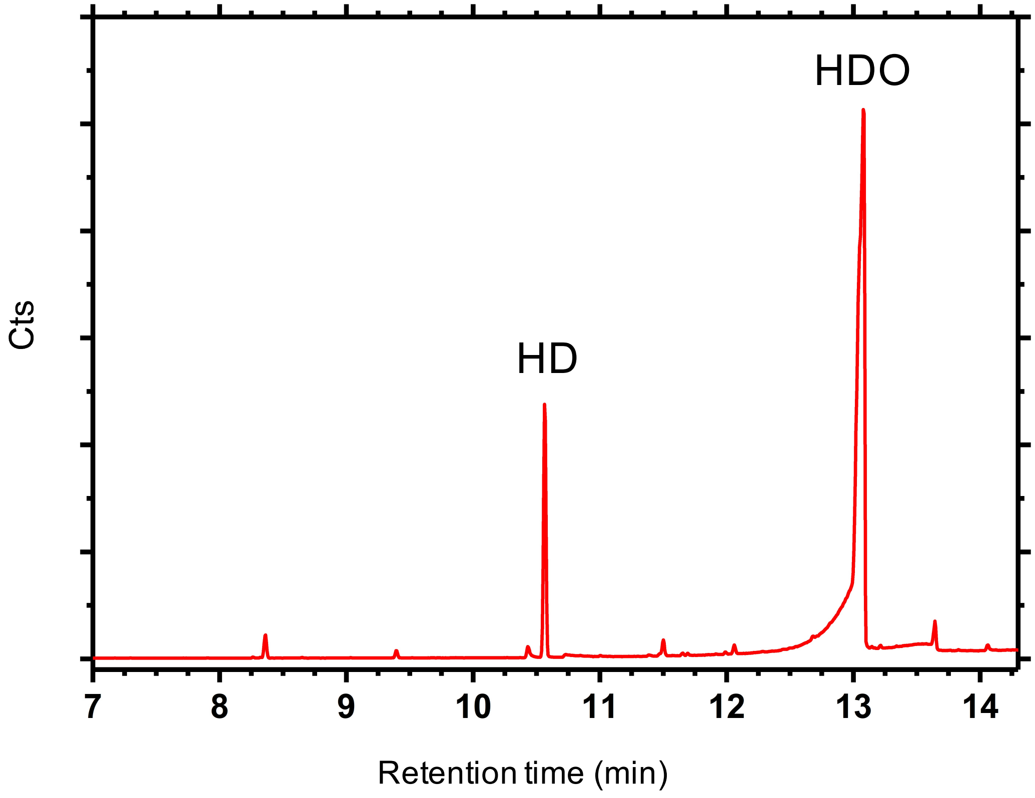

Supplementary Figure 24. GC-MS spectra immediately following the DRIFTS experiment of HD oxidation catalyzed by **SFC** (main text Figure 5). Conditions: **SFC** placed in a DRIFTS cup exposed to HD vapor in a gas stream of 2% relative humidity/Zero Air for 4 hours. GC-MS measurement taken via a 1.5 mL $CDCl_3$ extraction.

List of Abbreviations:

CEES	2-chloroethyl ethyl sulfide
CEESO	2-chloroethyl ethyl sulfoxide
$CEESO_2$	2-chloroethyl ethyl sulfone
DCB	dichlorobenzene
DRIFTS	diffuse reflectance infrared Fourier transform spectroscopy
EXAFS	extended X-ray absorption fine structure

FFT	fast Fourier transform
FID	flame ionization detector
GC	gas chromatography
GC-MS	gas chromatography-mass spectrometry
HD	bis(2-chloroethyl) sulfide (sulfur mustard)
HP	Hewlett Packard
Hz	hertz
IFEFFIT	library of XAFS algorithms
IR	infrared
MCT	mercury cadmium telluride
MeCN	acetonitrile
NMR	nuclear magnetic resonance
NSLS	national synchrotron light source
PTFE	polytetrafluoroethylene
QAS	quick X-ray absorption and scattering
RH	relative humidity
TBA	tetrabutylammonium
THT	tetrahydrothiophene
UV-vis	ultraviolet-visible
XAFS	X-ray absorption fine structure
XANES	X-ray absorption near edge structure
